# Activatable Silicon-Xanthene Photosensitizer for Photodynamic Therapy of Glioblastoma

**DOI:** 10.3390/pharmaceutics18040420

**Published:** 2026-03-29

**Authors:** Osman Karaman, Dilay Kepil, Mehrdad Forough, Zubeyir Elmazoglu, Gorkem Gunbas

**Affiliations:** 1 Department of Chemistry, Middle East Technical University, 06800 Ankara, Türkiye; karaman@metu.edu.tr (O.K.); dilay.kepil@metu.edu.tr (D.K.); mehrdadforogh@gmail.com (M.F.); 2 Department of Pharmacology, Ankara Medipol University, 06050 Ankara, Türkiye

**Keywords:** photodynamic therapy, glioblastoma, *β*-galactosidase, activatable PDT agents

## Abstract

**Background:** Photodynamic therapy (PDT) offers a promising complementary strategy for treating glioblastoma multiforme (GBM); however, limited control over photosensitizer activation and reduced efficacy under hypoxic conditions remain significant limitations. **Methods:** In this study, we present the synthesis and functional evaluation of **Gal-SiX**, an enzymatically activatable Si-xanthene-based activatable PDT agent designed to address these challenges. Prepared via an improved 10-step synthetic route, Gal-SiX exhibits clear turn-on fluorescence and absorbance responses upon β-galactosidase activation and efficiently generates reactive oxygen species in aqueous media. **Results:** Mechanistic studies revealed that **Gal-SiX** enables both Type I and Type II PDT pathways, a favorable feature for GBM environments characterized by restricted oxygen availability. In vitro assays conducted on U87MG glioblastoma cells and L929 healthy fibroblasts demonstrated light-dependent cytotoxicity, with IC_50_ values of 3.30 μM and 7.19 μM, respectively. **Gal-SiX** also showed minimal dark toxicity (>80 μM) and potent light-induced cytotoxicity, yielding a phototoxicity index of 24.8 in glioblastoma cells. Confocal imaging and MTT assays consistently confirmed enzymatic activation and effective PDT response at the cellular level. **Conclusions:** Overall, this work introduces the **first activatable Si-xanthene-based PDT agent** for glioblastoma and provides the first evidence that the Si-xanthene scaffold can support **dual Type I/II phototoxicity**. These results underscore **Gal-SiX**’s potential as a PDT platform for addressing the unique constraints of GBM biology.

## 1. Introduction

Cancer remains one of the foremost global health concerns, since effective treatment modalities for a range of cancer types are still elusive [1,2]. While significant progress has been made in cancer prevention, early detection, and treatment across various types of cancers, brain tumors persist as an unresolved clinical challenge [3,4]. Despite their lower incidence compared to many other types of cancer, brain tumors are among the most feared due to their high mortality rates [5]. Among the pediatric solid tumors, brain cancers are also the most prevalent and most fatal type of cancer. Furthermore, several adverse effects of medical operations such as surgery, chemotherapy, and/or radiotherapy are often observed in people who have suffered from a brain tumor and have survived into adulthood [6].

Among all types of brain tumors, glioblastoma is the most aggressive one, with a two-year survival rate below 35% [7]. In recent years, significant progress has been made in molecular profiling and genomic characterization, enhancing our understanding of the interdependence of glioblastoma proliferation and invasion. This has opened avenues for discoveries that may aid in differential diagnosis and novel treatment strategies [8]. Although several FDA-approved drugs have been developed for the treatment of glioblastoma, only three are currently used: Carmustine, Temozolomide, and Bevacizumab. Unfortunately, none of them has shown superiority over conventional chemotherapy and radiation in terms of extending survival [9]. The inadequacy of current treatment modalities for brain tumors emphasizes the urgent need for further research to develop more effective alternatives. In response to these limitations, a broad range of innovative therapeutic strategies for glioblastoma have been actively investigated, including molecularly targeted therapies, immunomodulatory approaches, nanotechnology-based delivery systems, and emerging physical treatment modalities. Recent reviews and experimental studies highlight both the promise and the persistent challenges of these strategies, particularly in overcoming tumor heterogeneity, therapeutic resistance, and the restrictive blood–brain barrier [10,11,12].

Photodynamic Therapy (PDT) is an alternative treatment method for diverse cancer types. It has gained attention due to its minimally invasive nature and fewer side effects compared to existing treatment methods [13,14] and holds potential for overcoming the barriers of brain cancer treatment. Nevertheless, its applicability remains limited to a small group of cancers because the requisite light does not penetrate human tissue well enough to trigger singlet oxygen generation, and it lacks selective targeting of cancer cells [15]. Another obstacle to PDT is the hypoxic nature of certain tumors, particularly glioblastomas. This characteristic feature of these tumors hinders the therapeutic efficacy of PDT [16]. To enhance PDT performance, numerous efforts have been made to increase the O_2_ concentration in the tumor area, including direct delivery of exogenous O_2_ to the tumor [17,18], in situ O_2_ generation [19,20], reduction in tumor O_2_ consumption by inhibiting respiration [21,22], regulation of the tumor microenvironment [23], and inhibition of hypoxia-inducible factor 1α (HIF-1α) signaling [24,25]. Furthermore, O_2_-independent or Type I-biased PDT, which relies more on radical formation than on classical singlet oxygen, is being actively explored as a way to bypass hypoxia-driven resistance [26]. Several PDT agents, including organic photosensitizers and metal complexes, have demonstrated potent photocytotoxicity against various cancer types [27,28,29,30,31,32,33,34,35,36,37,38,39]. However, studies focusing on hypoxic glioblastomas using Type I-based PDT agents remain scarce [40], and although targeted PDT approaches have been reported, no examples of activatable PDT agents have been described to the best of our knowledge [41].

Furthermore, designing an ideal photosensitizer poses another challenge for effective PDT. To achieve deep tissue penetration, the photosensitizer (PS) should absorb light within the therapeutic window (600–850 nm) [42,43,44]. Despite the development of numerous fluorophores in recent decades, only a small number have been modified into PSs due to the absence of absorption maxima within the therapeutic window, low aqueous solubility, and difficulties with structural modification [45,46,47,48]. The promising properties of xanthene-based dyes, such as water solubility, photostability, and ease of modification [49,50,51,52,53,54,55,56], motivated us to investigate improvements on classical xanthene dyes such as fluorescein and rhodamine towards the realization of red/near-infrared (NIR) absorbing/emitting dyes with highly cancer-selective activation for imaging and treatment opportunities. Previously, extending conjugation within the molecule has been a favored method for shifting absorption toward the NIR region [57,58]. This ultimately results in higher molecular weight and renders them ineffective for brain cancer treatment due to the highly selective nature of the blood–brain barrier (BBB) [59,60]. On the other hand, modification of certain parts of the xanthene core was shown to be highly effective in shifting the absorption maximum toward the NIR region without significantly affecting molecular weight [40,57,58]. In 2011, Nagano and colleagues introduced a red-shifted analogue of fluorescein by replacing the oxygen atom at the 10th position of the xanthene core with a silicon atom, resulting in a 100 nm red shift while preserving all the promising properties of classical fluorescein analogues [57]. To date, Si-Xanthenes has been used in numerous imaging studies; however, its applicability in PDT was demonstrated only recently by our group in our pursuit of viable PDT agents for brain cancer treatment [46,61,62,63,64]. Tetra-iodinated Si-Xanthene (SF-I) proved to be a theranostic agent, exhibiting significant toxicity toward various peripheral cancer cell lines. Nonetheless, the lack of an effective targeting moiety hinders its potential as a potent PDT agent [65].

*β*-Galactosidase (*β*-gal) is a lysosomal glycoside hydrolase that catalyzes the cleavage of *β*-D-galactosides into monosaccharides, playing a fundamental role in cellular catabolism and turnover of glycoconjugates and complex carbohydrates [66,67,68]. Under physiological conditions, *β*-gal activity is ubiquitous in lysosomes, but its expression and activity become significantly altered in various pathological states, particularly in the context of cancer biology and cellular senescence. One of the most widely studied phenomena involving *β*-gal in mammalian cells is senescence-associated *β*-galactosidase (SA-*β*-gal) activity, which is used as a canonical biomarker for senescent cells both in vitro and in tumor tissues [69]. Elevated *β*-gal activity has been observed in various malignancies, including ovarian, gastric, and other epithelial cancers [70,71], and this overexpression has been leveraged for selective imaging and detection of tumor and metastatic tissue using *β*-gal-activated probes [66,67]. Although abnormal *β*-gal levels are generally attributed to metastatic ovarian cancer [68], significantly elevated *β*-gal levels in gliomas compared to healthy cells have also been reported [72,73]. Due to its distinct cancer selectivity, the *β*-gal enzyme is utilized in fluorescent diagnostic studies [71,74,75]. However, *β*-gal enzyme-activatable phototherapeutic drugs remain surprisingly rare, with the first *β*-gal activatable PDT agent for brain cancers, using an iodinated resorufin photosensitizer, having been demonstrated by our group [76].

**Figure 1:**
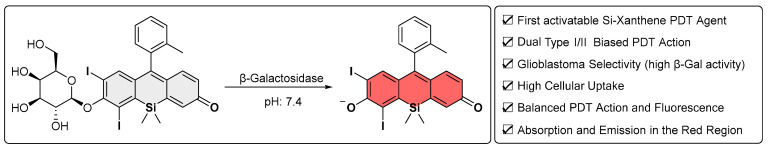
Structure of **Gal-SiX** and its conversion to **SiX** upon treatment with the enzyme.

Herein, we report the development of iodinated Si-Xanthene, modified with a *β*-gal-responsive *β*-D-galactopyranose moiety, as an activatable PDT agent (**Gal-SiX**). ***Gal-SiX*** demonstrated selectivity toward glioma (U87MG) cells over healthy (L929) cells in vitro. **Gal-SiX** remains in an OFF state until activation by *β*-gal and exhibits significantly low toxicity. Upon cleavage of the glycosidic linkage between the *β*-D-galactopyranose moiety and the photosensitizer (PS) in the presence of high *β*-gal levels in glioblastoma, the highly cytotoxic and moderately emissive iodinated Si-Xanthene core (**SiX**) is revealed (Figure 1). It is important to distinguish the present system from previously reported *β*-galactosidase-activated fluorescent probes developed primarily for cancer imaging and diagnostic applications, in which enzyme activation results solely in signal generation without therapeutic function. In parallel, recent aggregation-induced emission (AIE) and supramolecular PDT platforms have demonstrated enhanced phototoxicity through aggregation, confinement, or host–guest interactions rather than enzymatic bond cleavage [77,78,79,80]. In contrast, Gal-SiX relies on *β*-galactosidase-triggered chemical activation to release an active Si-xanthene photosensitizer capable of efficient ROS generation and light-induced cytotoxicity, thereby defining a distinct class of enzyme-activatable PDT agents and, to our knowledge, the first *β*-gal-responsive Si-xanthene-based PDT system evaluated for glioblastoma.

It is essential to note that **SiX** was not separately synthesized in this work; **SiX** refers to the released species after *β*-gal treatment of ***Gal-SiX***.

## 2. Materials and Methods

### 2.1. Chemistry

#### 2.1.1. General

The starting materials and solvents were purchased from Sigma-Aldrich, ABCR, TCI, and Merck. Solvents used for column chromatography, Hexane, ethyl acetate (EtOAc), and dichloromethane (DCM), were distilled prior to use over CaCl_2_. All reactions were conducted under a nitrogen atmosphere, unless otherwise specified. Reaction solvents (Diethyl ether, tetrahydrofuran (THF), DCM, Toluene, and dimethylformamide (DMF)) were directly used from the MBraun MBSPS5 (München, Bayern, Germany) solvent drying system. The reactions were monitored by thin-layer chromatography (TLC) (Merck Silica Gel 60 F254, Merck, Darmstadt, Germany) and visualized by UV light at 254 nm and 366 nm. Column chromatography of all products was performed using Merck Silica Gel (particle size: 0.040–0.063 mm, 230–400 mesh ASTM).

The structural analysis of the synthesized compounds was conducted using NMR and HRMS. ^1^H and ^13^C nuclear magnetic resonance spectra of the compounds were recorded in deuterated solvents with a Bruker Avance III Ultrashield 400 Hz NMR spectrometer (Bruker, Billerica, MA, USA, software: Bruker TopSpin version: 2.3). The chemical shifts were reported in parts per million (ppm) relative to tetramethylsilane (TMS) as an internal reference. High-Resolution Mass Spectra (HRMS) were acquired for the novel compounds using a Time-of-Flight mass analyzer with a Waters Synapt MS System (Wilmslow, UK). The purity of the final compound was confirmed by reverse-phase Agilent 1260 Infinity II High-Performance Liquid Chromatography (HPLC) System) (Agilent Technologies, Santa Clara, CA, USA, software: Agilent OpenLab CDS version 2.3.54) to be >95%.

The photophysical analyses were performed through absorption and fluorescence measurements. Absorption spectra were collected using a double-beam Jasco V-730 (JASCO Corporation, Easton, MD, USA) UV-vis spectrophotometer. A Cary Eclipse fluorescence spectrophotometer (Agilent Technologies, Santa Clara, CA, USA) equipped with a Cary single-cell Peltier temperature controller (Agilent Technologies, Santa Clara, CA, USA) was used to record the fluorescence emission spectra. All measurements were performed at room temperature using 10 mm quartz (3.5 mL, 111-QS, Hellma, Müllheim, Germany).

#### 2.1.2. Synthesis

**Compound 1:** In a 250 mL Schlenk tube, commercially available 3-bromoaniline (8.10 g, 47.1 mmol) and K_2_CO_3_ (18.88 g, 136.6 mmol, 2.9 eq.) were suspended with dry MeCN (110 mL) under nitrogen. BnBr (18.8 mL, 160.1 mmol, 3.4 eq.) was added slowly, and then the mixture was heated to 80 °C for 16 h. After the reaction was complete, MeCN was evaporated, and the crude product was treated with distilled water (80 mL) and extracted with EtOAc (2 × 80 mL). Collected organic phases were dried over Na_2_SO_4_, filtered, and the solvent was evaporated. The crude product was redissolved in EtOAc (5 mL) and then added dropwise to cold hexane (20 mL) to precipitate the product. After the addition was complete, the hexane/ethyl acetate mixture was removed, and the precipitate was dried under vacuum. The target product, compound **1**, was obtained as a white solid (13.6 g, **82**%). ^1^H NMR (400 MHz, CDCl_3_) δ 7.35 (t, *J* = 7.3 Hz, 4H), 7.29 (d, *J* = 7.1 Hz, 2H), 7.23 (d, *J* = 7.3 175 Hz, 4H), 7.01 (t, *J* = 8.1 Hz, 1H), 6.90 (s, 1H), 6.82 (d, *J* = 7.7 Hz, 1H), 6.64 (dd, *J* = 8.3, 2.0 Hz, 1H), 4.63 (s, 4H). ^13^C NMR (100 MHz, CDCl_3_) δ 150.6, 137.9, 130.6, 128.9, 127.2, 126.7, 123.6, 119.7, 115.15, 111.2, 54.1.

**Compound 2:** In a 250 mL Schlenk tube, compound **1** (4.85 g, 13.8 mmol, 2 eq.) and formaldehyde solution (2.07 g, 6.90 mmol, 30%) were added to AcOH (40 mL) and stirred until dissolved. Then the mixture was heated to 80 °C and stirred for 16 h. After that, the mixture cooled to room temperature and neutralized with satd. NaHCO_3_ and satd. NaOH solutions, then extracted with CHCl_3_ (2 × 50 mL). Collected organic phases were dried over Na_2_SO_4_, filtered, and the solvent was evaporated. The crude product was purified by column chromatography (silica, hexane: EtOAc, 6:1). The target product, compound **2**, was obtained as a white solid (4.05 g, **82**%). ^1^H NMR (400 MHz, CDCl_3_) δ 7.24 (t, *J* = 7.1 Hz, 8H), 7.18 (d, *J* = 6.5 Hz, 4H), 7.13 (d, *J* = 7.4 Hz, 8H), 6.88 (d, *J* = 2.0 Hz, 2H), 6.70 (d, *J* = 8.5 Hz, 2H), 6.47 (dd, *J* = 8.4, 2.2 Hz, 2H), 4.50 (s, 8H), 3.86 (s, 2H). ^13^C NMR (100 MHz, CDCl_3_) δ 148.8, 138.1, 131.1, 128.8, 127.4, 127.1, 126.7, 125.8, 125.8, 116.0, 111.8, 54.1, 39.9

**Compound 3:** In a 100 mL Schlenk tube, compound **2** (2.00 g, 2.79 mmol) was added to dry THF (12 mL) under nitrogen and cooled to −78 °C. Then, sec-BuLi (6.50 mL, 7.95 mmol, 1.4 M, 2.85 eq.) was added dropwise and stirred at that temperature for 1 h. 1.9 mL SiMe_2_Cl_2_ from stock solution ((stock solution: 1.9 mL SiMe_2_Cl_2_ + 3.8 mL THF), 5.30 mmol, 1.9 eq.) was added dropwise, then the reaction mixture was warmed to room temperature slowly. After reaching the room temperature, the mixture was stirred at that temperature for 3 h. After that, the reaction mixture was treated with 1 N HCl (1 mL), then neutralized with NaHCO_3_ (25 mL) and extracted with CHCl_3_ (2 × 50 mL). Collected organic phases were dried over Na_2_SO_4_, filtered, and the solvent was evaporated. The crude product was placed in a 100 mL two-necked round-bottomed flask, dissolved in acetone (26 mL), and cooled to 0 °C. To the reaction mixture, KMnO_4_ (1.22 g, 7.67 mmol, 2.75 eq.) was added in portions over 1.5 h, and the mixture was stirred for an additional 2.5 h at that temperature. After that, the reaction mixture was filtered through Celite and washed with CHCl_3_. The crude product was purified by column chromatography (neutral alumina, hexane: CHCl_3,_ 1:2). The target product, compound **3**, was obtained as a yellow solid (0.99 g, **57**%). ^1^H NMR (400 MHz, CDCl_3_) δ 8.28 (d, *J* = 9.0 Hz, 2H), 7.34–7.28 (m, 8H), 7.23 (d, *J* = 7.3 Hz, 12H), 6.86 (dd, *J* = 9.0, 2.7 Hz, 2H), 6.80 (d, *J* = 2.7 Hz, 2H), 4.71 (s, 8H), 0.14 (s, 6H). ^13^C NMR (100 MHz, CDCl_3_) δ 185.1, 150.7, 140.6, 137.8, 131.8, 130.4, 128.9, 127.3, 126.7, 115.2, 113.7, 54.2, −1.4.

**Compound 4:** In a Parr reactor, compound **3** (0.60 g, 1.0 mmol) was placed and dissolved with a MeOH: CHCl_3_ mixture (30 mL, 4:1). To this solution, Pd-C (0.12 g, 20% by mass) was added, and then air was removed by vacuum. The reactor was refilled with H_2_ gas (3.6 bar) and stirred for 3 days. After the reaction was complete, the mixture was filtered through Celite and washed with MeOH. The crude product was purified by column chromatography (silica, MeOH: DCM, 1:9). The target product, compound **4**, was obtained as a white solid (0.20 g, **77**%). ^1^H NMR (400 MHz, MeOD) δ 8.13 (d, *J* = 8.7 Hz, 2H), 6.87 (d, *J* = 2.4 Hz, 2H), 6.75 (dd, *J* = 8.7, 2.4 Hz, 2H), 0.40 (s, 6H). ^13^C NMR (100 MHz, MeOD) δ 187.6, 153.1, 142.8, 132.9, 131.1, 118.6, 116.7, −1.2.

**Compound 5:** In a 250 mL two-necked round-bottom flask, compound **4** (0.10 g, 0.4 mmol) was added and dissolved with MeOH: 6 N H_2_SO_4_ (92 mL, 1:1) under nitrogen and was cooled to 0 °C. Solution of NaNO_2_ (0.17 g, 2.5 mmol, 6.7 eq.) in H_2_O (4 mL) was added dropwise and stirred for 1 h at that temperature. After that, the mixture was added dropwise to the boiling 1 N H_2_SO_4_ (135 °C, 100 mL) over 1 h. Then it was stirred for 10 more minutes at that temperature before being cooled to room temperature. After the reaction was complete, the mixture was extracted with CHCl_3_ (2 × 50 mL). Collected organic phases were washed with brine, dried over Na_2_SO_4_, filtered, and the solvent was evaporated. The crude product was purified by column chromatography (silica, MeOH: DCM, 5:95). The target product, compound **5**, was obtained as a white solid (61 mg, **61**%). ^1^H NMR (400 MHz, MeOD) δ 8.22 (d, *J* = 8.8 Hz, 2H), 7.03 (d, *J* = 2.6 Hz, 2H), 6.91 (dd, *J* = 8.8, 2.6 Hz, 2H), 0.39 (s, 6H). ^13^C NMR (100 MHz, MeOD) δ 187.8, 162.3, 143.2, 133.9, 133.5, 120.2, 118.5, −1.4.

**Compound 6:** In a 250 mL two-necked round-bottom flask, compound **5** (100 mg, 0.37 mmol) and imidazole (260 mg, 3.83 mmol, 10.4 eq.) were added and dissolved with dry DCM (55 mL) under nitrogen. To this solution, solution of TBDMSCl (562 mg, 3.74 mmol, 10.1 eq.) in dry DCM (13 mL) was added slowly and stirred for 16 h at room temperature. After completion of the reaction, the mixture was treated with distilled water (50 mL) and extracted. Collected organic phases were washed with brine, dried over Na_2_SO_4_, filtered, and the solvent was evaporated. The crude product was purified with column chromatography (silica, hexane:DCM 1:8). The target product, compound **6**, was obtained as a white solid (153 mg, **83**%). ^1^H NMR (400 MHz, CDCl_3_) δ 8.38 (d, *J* = 8.7 Hz, 2H), 7.05 (d, *J* = 2.2 Hz, 2H), 6.99 (dd, *J* = 8.7, 2.5 Hz, 2H), 1.01 (s, 18H), 0.47 (s, 6H), 0.27 (s, 12H). ^13^C NMR (100 MHz, CDCl_3_) δ 186.1, 158.9, 141.3, 134.7, 132.4, 123.8, 121.9, 25.8, 18.4, −1.4, −4.2.

**Compound 7:** In a 100 mL two-necked round-bottom flask, commercially available o-bromotoluene (343 mg, 2.00 mmol, 5 eq.) was added to dry THF (30 mL) under nitrogen and cooled to −78 °C. After that, n-BuLi (0.80 mL, 2.0 mmol, 2.5 M, 5 eq.) was added dropwise and stirred for 30 min at that temperature. Then, compound **6** (200 mg, 0.40 mmol) in dry THF (5 mL) was added to the reaction mixture dropwise, and the mixture was allowed to slowly warm to room temperature and stirred for 1.5 h more at that temperature. After the completion of the reaction, the mixture was treated with 1 M HCl (12 mL) and extracted with DCM (50 mL). Collected organic phases were washed with brine, dried over Na_2_SO_4_, filtered, and the solvent was evaporated. The crude product was purified with column chromatography (silica, MeOH: CHCl_3_ 1:18). The target product, compound **7**, was obtained as a dark orange solid (86 mg, **63**%). ^1^H NMR (400 MHz, CDCl_3_) δ 7.38–7.34 (m, *J* = 7.4 Hz, 1H), 7.29 (d, *J* = 8.2 Hz, 2H), 7.09–7.04 (m, 3H), 6.94 (d, *J* = 9.4 Hz, 2H), 6.56 (dd, *J* = 9.4, 2.1 Hz, 2H), 3.49 (s, 1H), 2.03 (s, 3H), 0.43 (s, 3H), 0.41 (s, 3H). ^13^C NMR (100 MHz, CDCl_3_) δ 172.6, 161.5, 146.0, 140.5, 139.3, 135.9, 130.2, 129.9, 129.2, 129.0, 128.5, 125.7, 122.1, 19.6, −1.2, −1.5.

**Compound 8:** In a 100 mL two-necked round-bottom flask, compound **7** (69 mg, 0.20 mmol) and CsCO_3_ (326 mg, 1.00 mmol, 5 eq.) were added and dissolved with dry MeCN (45 mL) under nitrogen. After that, acetobromo-α-D-galactose (348 mg, 1.00 mmol, 5 eq.) was added and stirred for 16 h. After completion of the reaction, MeCN was removed under reduced pressure, and the mixture was dissolved in CHCl_3_ (50 mL), extracted with distilled water (50 mL), and washed with brine (50 mL). Collected organic phases were dried over Na_2_SO_4_, filtered, and the solvent was evaporated. The crude product was purified by column chromatography (silica, MeOH: CHCl_3,_ 1:18). The target product, compound **8**, was obtained as an orange solid (75 mg, **56**%). ^1^H NMR (400 MHz, CDCl_3_) δ 7.39–7.33 (m, 1H), 7.33–7.27 (m, 2H), 7.24 (d, *J* = 2.1 Hz, 1H), 7.06 (t, *J* = 6.5 Hz, 1H), 6.94 (d, *J* = 10.1 Hz, 1H), 6.85–6.76 (m, *J* = 9.2, 5.6 Hz, 3H), 6.22 (dd, *J* = 10.1, 1.9 Hz, 1H), 5.53–5.42 (m, 2H), 5.16–5.08 (m, 2H), 4.20–4.04 (m, 3H), 2.16 (s, 3H), 2.04 (dd, *J* = 6.7, 1.1 Hz, 6H), 2.00–1.97 (m, 6H), 0.46 (t, *J* = 6.0 Hz, 6H). ^13^C NMR (100 MHz, CDCl_3_) δ 184.4, 170.3, 170.2, 170.1, 169.4, 157.0, 157.0, 154.9, 146.8, 141.6, 140.6, 139.1, 137.2, 136.1, 136.0, 135.7, 135.0, 130.3, 130.2, 129.2, 128.4, 128.0, 125.9, 125.9, 122.9, 116.6, 98.2, 71.2, 70.7, 68.4, 66.7, 61.3, 61.3, 53.5, 20.8, 20.6, 20.6, 19.5, −1.2, −1.2, −1.4, −1.5.

**Compound 9:** In a 50 mL Schlenk tube, compound **8** (64 mg, 0.10 mmol) was added, and dissolved with EtOH (10 mL) under nitrogen. After that, I_2_ (48 mg, 0.19 mmol, 2 eq.) was added to the mixture and stirred for 15 min at 60 °C. Then, a solution of HIO_3_ (33.4 mg, 0.19 mmol, 2 eq.) in H_2_O (1.0 mL) was added dropwise to the reaction mixture, and the temperature was raised to 78 °C. The mixture was stirred for 2 h. After all starting material was consumed, EtOH was evaporated, and the mixture was treated with a 10% Na_2_S_2_O_3_ solution (10 mL) and extracted with EtOAc (50 mL). Collected organic phases were dried over Na_2_SO_4_, filtered, and the solvent was evaporated. The crude product was purified by column chromatography (silica, MeOH: DCM, 1:18). The target product, compound **9**, was obtained as a dark orange solid (75 mg, **85**%). ^1^H NMR (400 MHz, CDCl_3_) δ 7.74 (s, 1H), 7.41 (t, *J* = 7.2 Hz, 1H), 7.36–7.29 (m, 2H), 7.24 (s, 1H), 7.06 (t, *J* = 6.7 Hz, 1H), 6.80 (s, 2H), 5.50 (dd, *J* = 10.3, 8.0 Hz, 1H), 5.45 (d, *J* = 3.2 Hz, 1H), 5.18 (d, *J* = 7.7 Hz, 1H), 5.13 (dd, *J* = 10.4, 3.2 Hz, 1H), 4.21–4.08 (m, 3H), 2.17 (s, 3H), 2.06 (s, 3H), 2.04 (s, 3H), 2.02–1.98 (m, *J* = 4.8 Hz, 6H), 0.78 (t, *J* = 5.6 Hz, 6H). ^13^C NMR (100 MHz, CDCl_3_) δ 171.9, 170.1, 170.0, 169.9, 169.2, 158.1, 157.3, 151.4, 151.3, 143.0, 138.9, 135.6, 135.5, 134.2, 131.9, 130.3, 128.8, 128.8, 125.9, 123.0, 118.2, 116.6, 98.0, 98.0, 97.7, 71.2, 70.5, 68.3, 66.6, 61.2, 61.2, 20.6, 20.5, 20.4, 19.5, 19.5, −1.2, −1.2, −1.3, −1.3.

**Gal-SiX:** In a 25 mL Schlenk tube, compound **9** (75 mg, 0.1 mmol) was added, and dissolved with MeOH (7 mL) under nitrogen and cooled to 0 °C. To this solution, NaOMe (10 mg, 0.4, 4.4 eq.) in MeOH (1.1 mL) solution was added dropwise and stirred for 1 h at that temperature. After the reaction was complete, the mixture was neutralized with Amberlyst and then filtered. The collected filtrate was evaporated, and the crude product was purified with preparative HPLC. The target product, **Gal-SiX**, was obtained as a dark orange solid (57 mg, **93**%). ^1^H NMR (400 MHz, MeOD) δ 7.82 (s, 1H), 7.55–7.48 (m, *J* = 7.5, 5.1 Hz, 2H), 7.47–7.38 (m, *J* = 14.3, 7.1 Hz, 2H), 7.17 (d, *J* = 7.4 Hz, 1H), 7.05–6.99 (m, 1H), 6.90 (d, *J* = 9.1 Hz, 1H), 5.03 (dd, *J* = 7.7, 5.1 Hz, 2H), 3.91 (d, *J* = 3.3 Hz, 1H), 3.85 (dd, *J* = 9.5, 7.9 Hz, 1H), 3.79–3.74 (m, 2H), 3.62 (dd, *J* = 9.7, 3.2 Hz, 2H), 2.07 (s, 3H), 0.84 (dd, *J* = 5.3, 3.5 Hz, 6H). ^13^C NMR (100 MHz, MeOD) δ 172., 160.2, 160., 159.9, 152.7, 151.9, 143.0, 139.0, 136.0, 135.4, 132.7, 131.1, 130.0, 128.6, 125.6, 122.7, 122.6, 117.5, 117.4, 115.8, 100.4, 100.4, 99.9, 95.3, 75.9, 73.2, 70.5, 68.7, 61.0, 18.0, −2.6, −2.7, −2.7, −2.8. HRMS (ESI/MS) *m*/*z*: [M + H]+ Calcd. for C_22_H_18_I_2_O_2_Si^+^ 596.9200; Found 596.9240

#### 2.1.3. HPLC Analysis

Reverse-phase HPLC analyses were conducted using Agilent Technologies 1260 Infinity II series HPLC systems with a DAD detector. All analyses were performed using gradient elution with Milli-Q (0.1% TFA) (Merck Millipore, Darmstadt, Germany) and acetonitrile (0.08% TFA) as mobile phases, and the column compartment temperature was 40 °C. HPLC purifications were performed using a Phenomenex Kinetex (Phenomenex, Torrance, CA, USA) 5 μm C18 100 A semi-preparative column with a flow rate of 1.5 mL/min. For the purities, an Agilent Technologies Poroshell 120 EC-C18 analytical column was used at a flow rate of 0.5 mL/min.

### 2.2. Photophysical Characterization

#### 2.2.1. Enzymatic Activation

To demonstrate the enzymatic cleavage of the handle group of **Gal-SiX** in the presence of *β*-galactosidase, **Gal-SiX** (10 μM) in PBS buffer (pH 7.4, 1% DMSO) was incubated with *β*-galactosidase (5 U), and absorption spectra were recorded at 0, 10, 20, 30, 40, and 75 min. Time-dependent activation was observed, with complete activation achieved after 40 min of incubation.

#### 2.2.2. Fluorescence Quantum Yield

The samples’ fluorescence quantum yields were measured using a fluorescence spectrometer (FLS 1000, Edinburgh Instruments, Livingston, UK) equipped with an integrating sphere accessory. A continuous-wave xenon lamp served as the excitation source, and the emitted fluorescence was detected using a standard photomultiplier tube (PMT-900, Grandway Telecom Tech. Co., Shanghai, China) covering the wavelength range 200–800 nm. During measurements, the PMT was cooled to −20 °C using a built-in housing to reduce undesired dark current noise.

For quantum yield measurement, an integrating sphere (Edinburgh Instruments, Livingston, UK) was placed inside the spectrometer’s sample compartment. The internal cavity of the sphere was coated with a PTFE-like material, yielding reflectance >99% (>95%) over the wavelength ranges 400–1500 nm and 250–2500 nm, respectively. The sphere had two ports positioned 90° apart. The excitation beam was directed to the sample through the excitation port, and the fluorescence was collected from the emission port. The excitation port of the sphere included a lens to effectively focus the beam on the sample, while the emission port was an open aperture.

Prior to the experiments with the PSs, blank spectra were measured using the reference solvents (PBS, pH 7.4, 1% DMSO). For both measurements (blank and sample), two identical quartz cuvettes with equal volumes were used. Initially, the reference sample was placed inside the sphere, and the emission/excitation slits were adjusted to the excitation wavelength to ensure the PMT’s response remained linear during measurements. To cover a scattering range, the emission scans started 20 nm below the actual excitation wavelengths and ended at 900 nm. Additionally, the step size and integration time of the measurements were set to 1 nm and 0.2 s, respectively.

Upon completing all emission measurements of the samples and references, the quantum yields of the samples were determined using the Fluoracle software (version: 1.4.0). The built-in analysis tool calculates the quantum yield (QY) as:

*QY* = (*Es* − *EB*)/(*SB* − *Ss*),

where *Es* (*EB*) and *Ss* (*SB*) are the selected areas for the emitted and scattered signals of the sample (blank).

#### 2.2.3. Singlet Oxygen Trap Experiment

Singlet oxygen quantum yields were calculated using the following equation and methylene blue as the reference fluorophore according to our latest experimental procedure [41].

$$\phi_{\Delta sample}=\phi_{\Delta standard}\times (\frac{1-10^{-Astd}}{1-10^{-Asam}})\times (\frac{m_{sample}}{m_{standard}})$$

#### 2.2.4. General Procedure for Detection of ROS Type

To identify the type of reactive oxygen species (ROS) generated, several ROS-specific fluorescent probes were employed: Singlet Oxygen Sensor Green (SOSG) for the detection of singlet oxygen (^1^O_2_), Dihydrorhodamine 123 (DHR123) for superoxide anion (O_2_^•−^), and 2-[6-(4′-amino)phenoxy-3H-xanthen-3-on-9-yl]benzoic acid (APF) for hydroxyl radical (•·OH). The interaction between the probes and the generated ROS was assessed after photoirradiation of **Gal-SiX** in PBS buffer (pH 7.4) containing 1% DMSO, following incubation with *β*-galactosidase for 40 min at 37 °C.

Probe concentrations were maintained at 5 μM, and the activated **Gal-SiX** concentration was set at 10 μM. Fluorescence measurements were performed using a spectrofluorometer with emission wavelengths set at 525 nm for SOSG, 526 nm for DHR123, and 517 nm for APF. The cuvette containing the photosensitizer and the corresponding probe was irradiated with a 595 nm LED placed 10 cm away. Each irradiation lasted for 10 s, and the fluorescence intensity was monitored for up to 100 s.

#### 2.2.5. Interference Studies

To evaluate potential interference from biologically relevant molecules, various ions and small molecules—including sodium iodide (NaI), potassium chloride (KCl), lithium fluoride (LiF), calcium chloride (CaCl_2_), sodium nitrite (NaNO_2_), sodium thiosulfate (Na_2_S_2_O_3_), thiourea, hydrogen peroxide (H_2_O_2_), glucose, and homocysteine—were tested. Each was prepared at 1 mM in PBS and added to a solution of **Gal-SiX** (10 μM) in PBS buffer (pH 7.4, 1% DMSO), which was incubated with or without *β*-galactosidase (5 U). The absorbance of the resulting solutions was recorded.

### 2.3. In Vitro Experiments

#### 2.3.1. Cell Culture

Human glioblastoma cells (U87MG) and healthy cells (L929, mouse fibroblast cells) were used in this study. Cell lines were obtained from Dr. Safacan Kolemen Lab at Koc University. L929 mouse fibroblast cells were selected as the healthy control line because they are non-transformed, robust, and widely used for in vitro cytotoxicity testing under ISO 10993-5:2009 (Annex C: MTT cytotoxicity test) due to their reproducible growth and sensitivity [81]. Their use provides a standardized benchmark for comparative phototoxicity studies [82] and ensures methodological consistency when using U87MG cells under identical culture and irradiation conditions. Cells were cultured under standard conditions (in high-glucose DMEM supplemented with 10% FBS, 1% penicillin/streptomycin, 0.5% amphotericin B, and 2 mM glutamine at 37 °C with 5% CO_2_). The cells were passaged every 3–4 days when they reached 80–90% confluency. **Gal-SiX** was dissolved in cell culture-grade DMSO and further diluted in complete medium for experimental use.

#### 2.3.2. Photodynamic Therapy

For photodynamic therapy studies, cells were incubated with increasing concentrations of **Gal-SiX** (0–10 μM) in the dark for 0.5–2 h, then irradiated with LED light (595 nm, 8.12 mW/cm^2^) for 2 h. The incubation medium was not replaced before irradiation to maintain continuous *β*-galactosidase-mediated activation of **Gal-SiX** throughout the incubation period. This approach preserves both intracellular and extracellular activation kinetics, ensuring accurate assessment of the agent’s full photodynamic potential under enzyme-active conditions and reflecting the in vitro microenvironment of solid tumors, where limited diffusion and sustained enzymatic activity allow local accumulation of the activated PS [70,83,84,85]. After the irradiation period, cells were further incubated in the dark for 24 h to evaluate phototoxic effects. In parallel experiments to determine indices, U87MG and L929 cells were treated with increasing concentrations of **Gal-SiX** (0.1–160 μM) for 24 h in the dark, followed by viability assessment using the MTT assay. Phototoxicity index (PI), selectivity index (SI), and in vitro therapeutic index (TI) values were calculated as follows:

**PI** = IC_50, dark_/IC_50, light_                        

**SI** = IC_50, light (L929)_/IC_50, light (U87MG)_

**TI** = IC_50, dark (L929)_/IC_50, light (U87MG)_

#### 2.3.3. Cell Viability Analysis

Cells were seeded at 5 × 10^4^ cells/well on a flat-bottom 96-well plate in complete medium for 24 h. After incubation, cells were treated with **Gal-SiX** as described above. Following the experimental protocol, cells were treated with 0.5 mg/mL MTT in fresh medium for 2–4 h at 37 °C. The formazan crystals were dissolved with 10% SDS in PBS (0.01 N HCl) and incubated at 37 °C overnight. Absorbance values of each well were measured at 490 nm and 570 nm wavelengths using a MultiskanSky Microplate Reader (Thermo Scientific, Waltham, MA, USA). The results were expressed as percentages relative to the control samples treated with DMSO (0.2%) (*n* = 6–8). IC_50_ values were determined using concentration-normalized response curves derived from non-linear regression analysis (GraphPad Prism 9.02, GraphPad Software Inc., San Diego, CA, USA).

#### 2.3.4. Cellular Internalization and Activation

For cellular internalization and activation studies, U87MG and L929 cells were seeded in black 24-well plates (2 × 10^4^ cells/well) and incubated overnight. Cells were treated with **Gal-SiX** (1 µM) for 0.5, 1, 2, and 4 h, then washed twice with 1× PBS. Cells were stained with Hoechst 33,342 (0.5 µg/mL) for 15 min in serum-free medium at 37 °C. After washing steps, cells were fixed with 4% paraformaldehyde for 15–20 min at RT. Confocal images were captured using a Zeiss LSM 900 CLSM (Carl Zeiss Microscopy GmbH, Baden-Württemberg, Germany) at a 40× objective with constant exposure parameters. The fluorescence intensities of each time point were normalized relative to those of the 0.5 h L929 cells to evaluate the time-dependent activation and internalization states. Each experimental condition was performed in triplicate (*n* = 3). D-galactose (competitive inhibitor for *β*-galactosidase) was used to evaluate the cage-cleavage of **Gal-SiX**. In brief, cells were pre-treated with the increasing concentrations (5–50 mM) of D-galactose (in PBS) for 4 h, followed by **Gal-SiX** (2.5 µM, 1 h) post-treatment and PDT application. After 24 h of dark incubation, MTT analysis was performed (*n* = 6).

#### 2.3.5. Subcellular Co-Localization Experiments

Cells were seeded in 35 mm glass-bottom confocal dishes (2 × 10^4^ cells/well) and treated with **Gal-SiX** (1 µM and 2.5 µM) for 1 h. After incubation periods, cells were washed with PBS and incubated with the following organelle-specific trackers: MitoTracker™ Green FM (100 nM, 45 min, Thermo Scientific, Waltham, MA, USA), LysoTracker™ Yellow HCK-123 (75 nM, 45 min, Thermo Scientific, Waltham, MA, USA), ER-Tracker™ Green (BODIPY™ FL Glibenclamide, Thermo Scientific, Waltham, MA, USA) (1 µM, 30 min), and Hoechst 33,342 (0.5 µg/mL, 15 min, Hoechst AG, Höchst, Germany). Images were captured under the conditions with specified excitation/emission wavelengths for Hoechst, 361/497 nm; MitoTracker, 488/516 nm; LysoTracker, 465/535 nm; ER-Tracker, 504/511 nm; **Gal-SiX**, (Alexa 594 filter; 570–620 nm, Thermo Scientific, Waltham, MA, USA). The fluorescence intensities of the photosensitizers were kept consistent by adjusting the image-processing parameters uniformly. Co-localization was evaluated by calculating Pearson correlation coefficients (PCCs) using the co-localization analysis tool in Zeiss Zen Blue (Carl Zeiss Microscopy GmbH, Baden-Württemberg, Germany), comparing red-channel intensity with organelle-specific green signals. All experimental conditions were conducted in triplicate (40× and 63×, *n* = 3).

#### 2.3.6. Scavenger Assays

Reactive oxygen species (ROS) generation was evaluated using the DCFH-DA (2′,7′-dichlorofluorescin diacetate) probe using a Zeiss LSM 900 CLSM confocal microscopy (Carl Zeiss Microscopy GmbH, Baden-Württemberg, Germany). Briefly, U87MG glioblastoma cells were seeded in 96-well plates at 3 × 10^5^ cells/well and allowed to adhere overnight under standard culture conditions. The cells were then treated with **Gal-SiX** at its IC_50_ concentration for 1 h in the dark, followed by irradiation with LED light (595 nm, 8.12 mW/cm^2^) for 2 h. To investigate the contribution of different ROS types, parallel groups were co-treated with specific ROS scavengers added 1 h prior to photosensitizer administration. The scavengers and their working concentrations were as follows: N-acetylcysteine (NAC, 5 mM) thiol-based ROS scavenger; sodium azide (NaN_3_, 5 mM) and histidine (5 mM) for singlet oxygen (^1^O_2_); Tiron (100 μM) for superoxide anion (O_2_^•−^); mannitol (25 mM) for hydroxyl radicals (HO**•**); and Trolox (25 μM) for peroxyl radicals (ROO**•**).

For imaging, cells were washed twice with PBS and incubated with DCFH-DA (20 μM) and Hoechst 33,342 (0.5 μg/mL) in serum-free medium for 20–30 min at 37 °C. After staining, cells were rewashed with PBS, and fresh serum-free medium was added. Fluorescence imaging was performed using a Zeiss LSM 900 confocal laser scanning microscope (ZEISS ZEN, Oberkochen, Germany) at 488/535 nm (ex/em) for DCF and 361/497 nm (ex/em) for Hoechst, using a 10× objective. Representative fields from each condition were captured to assess intracellular ROS levels and the effect of scavengers. All experiments were performed in biological triplicate (*n* = 6). For viability assessment, cells were incubated in the dark for an additional 24 h under standard conditions after the scavenger assay protocol, then subjected to MTT analysis.

#### 2.3.7. Intracellular Type I ROS Detection

The DCF probe was used to detect intracellular general reactive oxygen species. The DHR123 probe was employed to monitor hydrogen peroxide, hypochlorous acid, and mitochondrial ROS. The DHE probe was used specifically to detect superoxide anion (O_2_^•−^). The APF probe was used to evaluate hydroxyl radical (HO**•**), hypochlorous acid (HOCl), and peroxynitrite anion (ONOO^−^). After incubation with **Gal-SiX** (2.5 μM) for 1 h in the dark, the medium was removed, and cells were subsequently stained with DCF (10 μM), DHR123 (5 μM), DHE (5 μM), or APF (10 μM) in fresh medium for an additional 30 min at 37 °C. Cells were then irradiated with or without 595 nm LED light (8.12 mW/cm^2^) to evaluate ROS production under both light-exposed and dark conditions. Following irradiation, cells were washed twice with PBS, counterstained with Hoechst 33,342 (0.5 μg/mL) for 15 min to visualize the nuclei, and then washed again with PBS. Confocal fluorescence imaging was performed using a 40× objective with the following excitation/emission settings: 488/535 nm for DCF, 508/536 nm for DHR123, 535/610 nm for DHE, 490/515 nm for APF, and 361/497 nm for Hoechst. No background fluorescence was observed under these conditions (*n* = 3).

#### 2.3.8. Acridine Orange/Ethidium Bromide (AO/EtBr) Dual Staining

Acridine orange/ethidium bromide (AO/EtBr) dual staining was performed to distinguish live, apoptotic, or necrotic cells based on differential membrane permeability. In brief, U87MG and L929 cells were plated in a 48-well plate at 5 × 10^4^ cells/well and treated with an IC_50_ dose of **Gal-SiX**. After the irradiation period, cells were incubated at 37 °C for 20 min and then stained with AO (2.5 μg/mL)/EtBr (2.5 μg/mL) in serum-free DMEM for an additional 20–30 min in the dark. After washing in 1× PBS, confocal images were acquired at 500 nm/525 nm (ex/em) and 530 nm/617 nm (ex/em) for the AO and EtBr dyes, respectively. (10×, *n* = 6)

#### 2.3.9. TBARS Assay for Lipid Peroxidation

The impact of **Gal-SiX** on cellular lipid peroxidation was assessed using the thiobarbituric acid reactive substances (TBARS) assay, which quantifies malondialdehyde (MDA), a stable end-product of lipid oxidation [86]. Cells were seeded in 6-well plates at a density of 1 × 10^6^ cells per well and allowed to adhere overnight. Subsequently, the PS was applied at an IC_50_ concentration following the established PDT protocol. Lipid peroxidation was evaluated immediately after irradiation and after a 24 h post-treatment period. At each time point, cells were collected and lysed in RIPA buffer (Merck, Darmstadt, Germany). Lysates were then processed according to the TBARS procedure. Briefly, samples were mixed with 0.8% thiobarbituric acid prepared in 20% trichloroacetic acid, incubated at 95 °C for 2–2.5 h, and the absorbance of the resulting chromogenic product was measured at 532 and 555 nm using a spectrophotometer (Thermo, Dresden, Germany). Results were expressed as percentages relative to untreated controls and normalized for protein content and cell viability (*n* = 3–4).

#### 2.3.10. Determination of Free Unsaturated Lipid Content

Intracellular lipid accumulation following **Gal-SiX** treatment was quantified by the sulfo-phospho-vanillin assay, which primarily detects unsaturated free lipids [87]. Cell lysates were prepared as described in the lipid peroxidation experiment. Protein concentration was determined using the bicinchoninic acid (BCA) method (Thermo Scientific, Dresden, Germany). For analysis, 40 µL of each lysate was combined with 200 µL of concentrated H_2_SO_4_, incubated at 90 °C for 10 min, and then cooled to room temperature. Subsequently, 120 µL of vanillin solution (1 mg/mL in 17% phosphoric acid) was added, producing a red chromophore that was quantified spectrophotometrically at 520–540 nm. Data were normalized to protein concentration and expressed as a percentage of the untreated control group (*n* = 3–4).

#### 2.3.11. Intracellular Thiols Detection

Cellular reduced thiol levels (-SH) were quantified using a colorimetric method according to the manufacturer’s instructions, which relies on the reaction of -SH with Ellman’s reagent (5,5′-dithiobis-2-nitrobenzoic acid, DTNB) (Thermo Scientific, Waltham, MA, USA) to generate a yellow-colored 2-nitro-5-thiobenzoate product [88]. Cell lysates were prepared as described previously, and Ellman’s reagent (200 μM) was freshly prepared in 1× PBS. Equal volumes of lysate (100 μL) and reagent (100 μL) were mixed and incubated for 30 min at room temperature in the dark. The absorbance of the reaction product was measured at 412 nm using a microplate reader (Thermo, Dresden, Germany). Free thiol levels were calculated as percentages relative to untreated control cells and normalized to protein concentration (*n* = 3–4).

#### 2.3.12. Statistical Analysis

Statistical analyses were performed using GraphPad Prism 9.02 (GraphPad Software Inc.). For multiple group comparisons, the Kruskal–Wallis test was employed, followed by Dunn’s post hoc test. All data are presented as mean ± standard deviation (SD). A *p* value of *p* < 0.05 was considered statistically significant.

## 3. Results and Discussion

### 3.1. Synthesis of ***Gal-SiX***

**Scheme 1:**
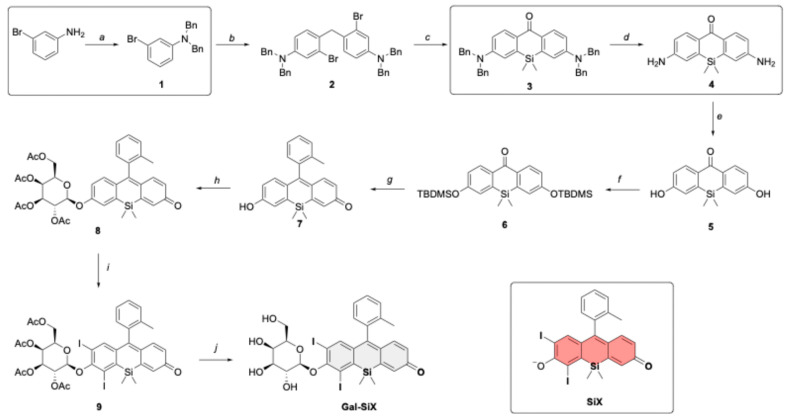
Synthetic route for **Gal-SiX**. a. K_2_CO_3_, BnBr, MeCN, 80 °C,16 h **82**%. b. Formaldehyde, AcOH, 80 °C,16 h, **82**%. c. 1-sec-BuLi, Et_2_O, −78 °C, 1 h, SiMe_2_Cl_2_, −78 °C 2-KMnO_4_ Acetone 0 °C, 4 h **57**%. d. H_2_, Pd-C, 3.6 bar CHCl_3_-MeOH (1:4) 3 d, **77**%. e. 1-MeOH-6 N H_2_SO_4_, NaNO_2_ in H_2_O, 0 °C, 1 h, 2–1 N H_2_SO_4_, 135 °C, 1 h, **61**%. f. imidazole, TBDMSCl, DCM, 1 d, rt, **83**%. g. *o*-bromotoluene, n-BuLi, THF, −78 °C, then **7**, **63**%. h. acetobromo-α-D-galactose, Cs_2_CO_3_, MeCN, 24 h, **56**%. i. I_2_, HIO_3_, EtOH, 78 °C, 2 h, **85**%. j. NaOMe, MeOH, 0 °C, 1 h, **90%**. Structure of *β*-Gal cleaved **Gal-SiX, SiX**.

The synthesis of **Gal-SiX** was achieved via a 10-step process by modifying the original synthetic route of Tokyo Magenta (TM) developed by Nagano et al. [57] (Scheme 1) This modification incorporated a greener approach by changing the protecting groups for the aniline moieties and using catalytic amounts of palladium for the deprotection, rather than stoichiometric quantities of the palladium reagent (Scheme 1, boxed reactions).

The synthetic pathway for **Gal-SiX**, as shown in Scheme 1, begins with the protection of commercially available 3-bromoaniline using a benzyl protective group, which results in the formation of a dibenzyl-protected aniline derivative, compound **1**, with an 82% yield. Compound **1** is then condensed with formaldehyde to establish a carbon bridge within the xanthene core. After successful synthesis of compound **2**, the dimethylsilane moiety is introduced into the core via a lithium-halogen exchange reaction with sec-BuLi. Once the silicon bridge is established, the crude product is dissolved in acetone, and KMnO_4_ is used to oxidize the dibenzylic position, yielding compound **3**. The deprotection of the benzyl groups proved challenging. Initial efforts to remove the four benzyl groups via hydrogenation at 1 atmosphere of pressure required 6 days to complete. Implementing the reaction in a Parr reactor with 3.6 bar H_2_ reduced the reaction time to three days while maintaining a comparable yield. After completing the optimization studies for the synthesis of compound **4**, the Sandmeyer reaction was used to obtain compound **5**. The hydroxyl groups were then protected with TBDMS to give compound **6** with high yield. Following the synthesis of the Si-xanthone core, commercially available *o*-bromotoluene was introduced at the 9th position of the Si-xanthone core, yielding compound **7**, TM. TM has been widely used in numerous imaging studies due to its exceptional photophysical properties [89,90,91,92]. Compound **7** was further modified with a *β*-galactose derivative to function as an activatable PDT agent. Heavy-atom incorporation into the PS was achieved by iodinating compound **8** with molecular iodine in the presence of HIO_3_, yielding a high yield. Finally, the desired PS, **Gal-SiX**, was obtained by removing the acetyl protective groups from the *β*-Gal moiety. The synthesized PS, **Gal-SiX**, was characterized using ^1^H, ^13^C NMR, and HRMS (Figures S29–S31). Before investigating its photophysical properties and conducting in vitro cell studies, **Gal-SiX** was purified by reverse-phase HPLC to achieve greater than 95% purity (Figure S1).

### 3.2. Optical Characterization

**Figure 2:**
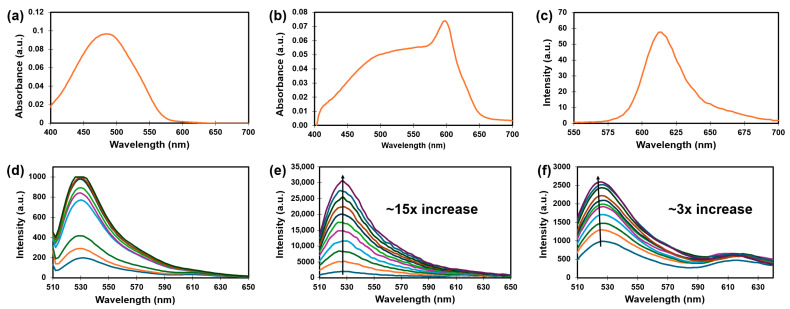
(**a**) Absorption spectrum of **Gal-SiX** (10 μM) in PBS buffer (pH 7.4, 1% DMSO). (**b**) Absorption spectrum of ***Gal-SiX*** (10 μM) in PBS buffer (pH 7.4, 1% DMSO) upon incubation with *β*-galactosidase (5 U) for 40 min (37 °C). (**c**) Fluorescence spectrum of **Gal-SiX** (10 μM) in PBS buffer (pH 7.4, 1% DMSO) upon incubation with *β*-Galactosidase (5 U) for 30 min (37 °C). (**d**) Fluorescence spectra of SOSG (5 μM) containing **Gal-SiX** (10 μM), (**e**) Fluorescence spectra of DHR 123 (5 μM) containing **Gal-SiX** (10 μM) and (**f**) Fluorescence spectra of APF (5 μM) containing **Gal-SiX** (10 μM) in PBS buffer (pH: 7.4, 1% DMSO) upon incubation with *β*-galactosidase (5 U) for 40 min (37 °C) irradiated with LED light (595 nm, 100 s total irradiation time, 3.50 mW/cm^2^) to detect ^1^O_2_ generation efficiency (**d**), to detect O_2_^•−^ generation efficiency (**e**), to detect •OH generation efficiency (**f**).

First, the absorption and fluorescence spectra of **Gal-SiX** were recorded before *β*-galactosidase enzyme addition in PBS buffer (pH 7.4, 1% DMSO), and **Gal-SiX** exhibited an absorption maximum at 486 nm (Figure 2a) and showed no detectable emission.

Upon incubation of **Gal-SiX** with *β*-galactosidase (5 U) for 40 min (37 °C), a red-shifted absorption maximum centered at 598 nm, which belongs to the **SiX** (Scheme 1), was obtained, accompanied by fluorescence emission with a maximum at 613 nm (Figure 2b,c). It is important to note that some unreacted **Gal-SiX** may be responsible for the broad absorption observed. Φ_F_ was calculated as 6.4% for **SiX** by employing Tokyo Magenta as a reference dye [54], which is significantly lower compared to Tokyo Magenta, as expected, due to intersystem crossing (ISC) by the heavy atom effect (Table 1). To demonstrate the ex vivo activation of **Gal-SiX** upon incubation with *β*-galactosidase, **Gal-SiX** (10 μM) in PBS buffer (pH 7.4, 1% DMSO) was incubated with *β*-galactosidase (5 U), and absorption spectra were recorded at various time intervals (0–75 min). The maximum activation was observed after 40 min of incubation (Figure S2), and subsequent photophysical measurements were performed following 40 min of incubation with *β*-galactosidase (5 U). **Gal-SiX** was further treated with biologically relevant analytes, with or without *β*-galactosidase, to assess selectivity for this enzyme. No significant changes were recorded in the absorption spectra of **Gal-SiX** in the presence of these analytes, indicating high selectivity for *β*-galactosidase (Figure S6).

Before conducting ROS generation measurements, the photostability of **Gal-SiX** and **SiX** was evaluated under irradiation with a 595 nm LED light source at a distance of 10 cm (3.50 mW/cm^2^). After 60 min of dark incubation in cell culture medium, with or without *β*-galactosidase, the absorption spectra of **Gal-SiX** and **SiX** were recorded at various time points up to 2 h of light exposure. To our delight, no significant change in the absorption maxima was observed for **Gal-SiX** and the cleaved active form **SiX** (Figure S3a–d). Subsequently, the singlet oxygen (^1^O_2_) generation capacity of **SiX** was investigated using the commonly employed ^1^O_2_ trap molecule, 2,2′-(anthracene-9,10-diyl)bis(methylene)dimalonic acid (ADMDA). Upon irradiation of *β*-Gal-treated **Gal-SiX** (**SiX**) with an LED at 595 nm, a time-dependent decrease in the absorption peak of ADMDA at 380 nm was observed due to cycloaddition reaction between photosensitized ^1^O_2_ and the anthracene ring (Figures S4 and S5). In contrast, no change in the absorption signal was observed when **Gal-SiX** was irradiated in the absence of the enzyme, as the photosensitizer cannot absorb the excitation light under these conditions (Figure S3a). The singlet oxygen quantum yield for **SiX** was calculated to be 52% (Table 1) using methylene blue as a reference (Φ_Δ_ = 0.52 in PBS buffer) [93]. To further support ^1^O_2_ generation, **SiX** was illuminated in the presence of the singlet oxygen sensor green (SOSG), and a notable increase in SOSG emission (Figure 2d) confirmed successful ^1^O_2_ generation.

To further evaluate the ROS generation capacity of **Gal-SiX**, the PS was incubated with *β*-galactosidase (5 U) for 40 min at 37 °C and treated with dihydrorhodamine 123 (DHR123) and 2-[6-(4′-amino)phenoxy-3H-xanthen-3-on-9-yl]benzoic acid (APF) to investigate its superoxide and other Type I ROS (peroxyl, hydroxyl, hypochlorite anions) generation capabilities. (Figure 2d) A marked enhancement in the fluorescence (15-fold) of oxidized DHR123 supported a strong ROS production through the Type I mechanism. (Figure 2e) Additionally, a slight increase in APF emission suggested a measurable hydroxyl radical (•OH) and peroxynitrite (ONOO^−^) generation capacity for **SiX** (Figure 2f).

### 3.3. In Vitro Studies

#### 3.3.1. Cytotoxicity Analysis

**Figure 3:**
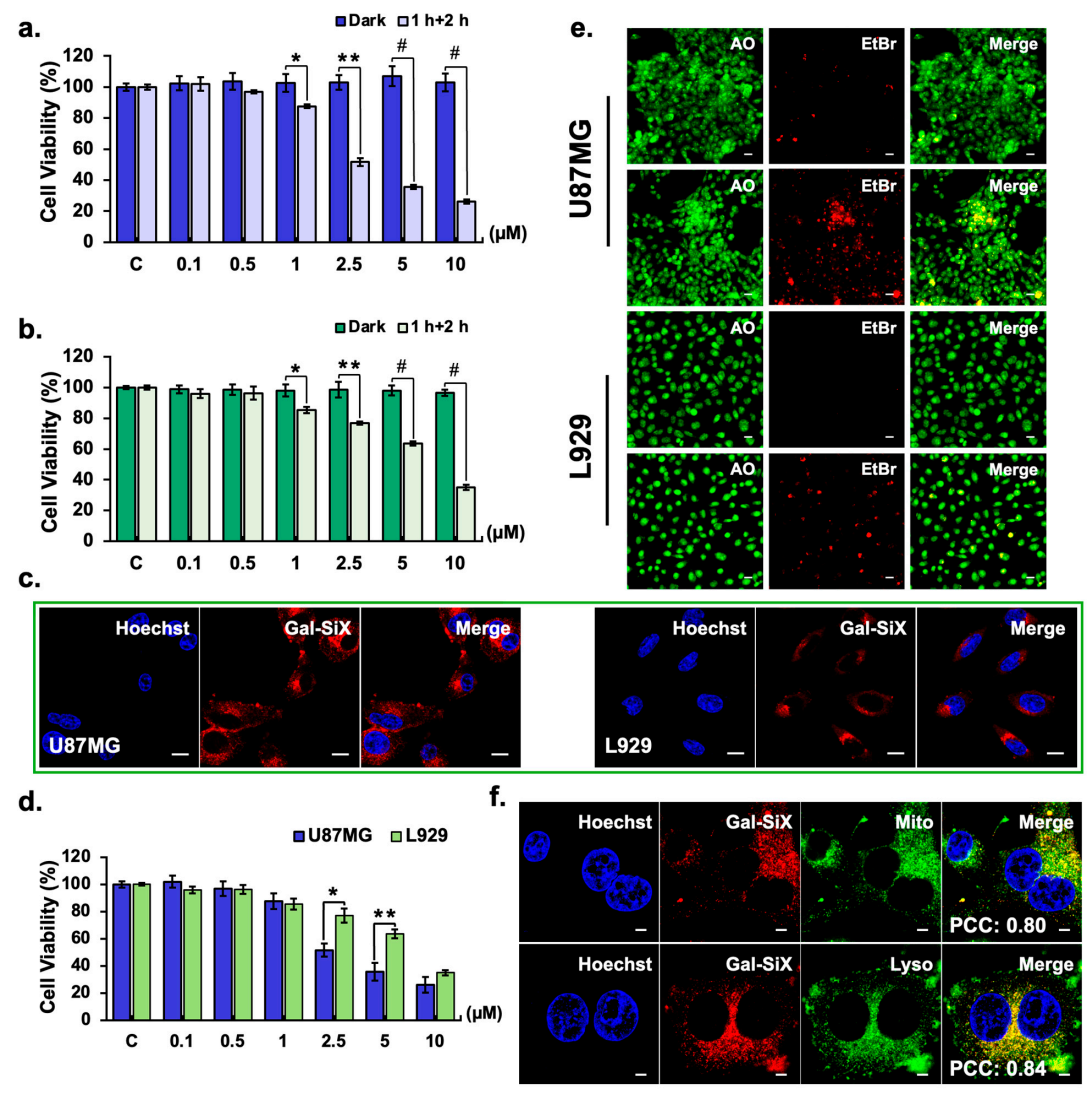
In vitro evaluation of phototoxicity, cellular localization, and cell death mechanisms of **Gal-SiX**. Cell viabilities of U87MG (**a**) and L929 (**b**) cells after 1 h incubation with **Gal-SiX** (0.1–10 μM), followed by 2 h LED irradiation (595 nm, 8.12 mW/cm^2^) and 24 h rest in the dark. (**c**) Confocal images depicting cellular internalization and activation state of **Gal-SiX** (1 μM, 1 h incubation) in U87MG and L929 cells. (**d**) Comparative cell viability of U87MG and L929 cells after **Gal-SiX** (0.1–10 μM, 1 h incubation) treatment under 2 h LED irradiation (595 nm, 8.12 mW/cm^2^), and 24 h dark incubation (*n* = 6). (**e**) Representative confocal images showing AO/EtBr dual staining in U87MG and L929 treated with **Gal-SiX** (IC_50_, 1 h incubation), followed by 2 h LED irradiation (595 nm, 8.12 mW/cm^2^). Green: Acridine orange (AO), live cells; Red: Ethidium bromide (EtBr), late apoptotic or necrotic cells (*n* = 6, Scale bar: 20 µm). (**f**) Subcellular localization of **Gal-SiX** (1 μM, 1 h incubation) (Red) with mitochondria (Green: MitoTracker) and lysosomes (Green: LysoTracker) in U87MG cells. Nuclei stained with Hoechst 33,342 (blue). (*n* = 3) Scale bar: 5 μm. * *p* < 0.05, ** *p* < 0.01, ^#^ *p* < 0.001 vs. untreated control (C).

The phototoxicity of **Gal-SiX** was systematically evaluated in U87MG glioblastoma and L929 healthy cells. To determine the optimal incubation and light exposure conditions, cells were first incubated with increasing concentrations (0.5–10 μM) of **Gal-SiX** for 0.5, 1, and 2 h in the dark, followed by LED irradiation at 1 cm over the lid (595 nm, 8.12 mW/cm^2^) for 0.5, 1, or 2 h, and subsequent incubation in the dark for 24 h (Figure S7). In U87MG cells, a more pronounced reduction in cell viability was observed with longer light exposure times, particularly at higher concentrations and after 1 or 2 h pre-incubation periods. In contrast, L929 cells exhibited relatively higher viability under similar conditions, indicating lower phototoxic sensitivity. Based on these optimization results, a protocol consisting of 1 h pre-incubation followed by 2 h LED irradiation was selected for further studies. Under these optimized conditions, U87MG cells demonstrated a concentration-dependent decrease in cell viability upon light activation, with significant effects observed at concentrations ≥1 μM (Figure 3a). In contrast, dark-treated cells maintained high viability even at the highest tested concentrations, confirming minimal dark toxicity. Similar trends were observed in L929 cells; however, the overall phototoxicity was less pronounced than in U87MG cells (Figure 3b). The calculated IC_50_ values (at optimized conditions) were 3.3 ± 0.1 μM for U87MG cells and 7.19 ± 0.32 μM for L929 cells. IC_50_ values for the other conditions were presented in Supplementary Table S2. The photodynamic performance and tumor selectivity of **Gal-SiX** were quantitatively evaluated using established metrics, including the phototoxicity index (PI), selectivity index (SI), and in vitro therapeutic index (TI). **Gal-SiX** exhibited high dark IC_50_ values of 81.92 ± 2.94 μM in U87MG and 116.6 ± 3.34 μM in L929 cells, confirming its minimal dark cytotoxicity. Under light irradiation, these values translated to a PI of 24.8 and an SI of 2.18, indicating that tumor cells were approximately two-fold more susceptible to **Gal-SiX** under light conditions than healthy fibroblast cells. Furthermore, the calculated TI (35.3) underscores the broad therapeutic window of **Gal-SiX**, indicating that high concentrations safely tolerated by normal cells in the dark are sufficient to induce potent, light-activated phototoxicity in U87MG cells (Table S3).

#### 3.3.2. Cellular Uptake and Activation-Induced Cell Death

To establish the correlation between internalization and the observed cytotoxicity, the intracellular uptake and activation kinetics of **Gal-SiX** were further investigated in U87MG and L929 cells at different dark incubation periods (0.5, 1, 2, and 4 h) with confocal microscopy (Figure S8). In U87MG cells, a progressive increase in red fluorescence intensity was observed with more extended incubation periods, indicating time-dependent internalization and activation of **Gal-SiX**.

**Figure 4:**
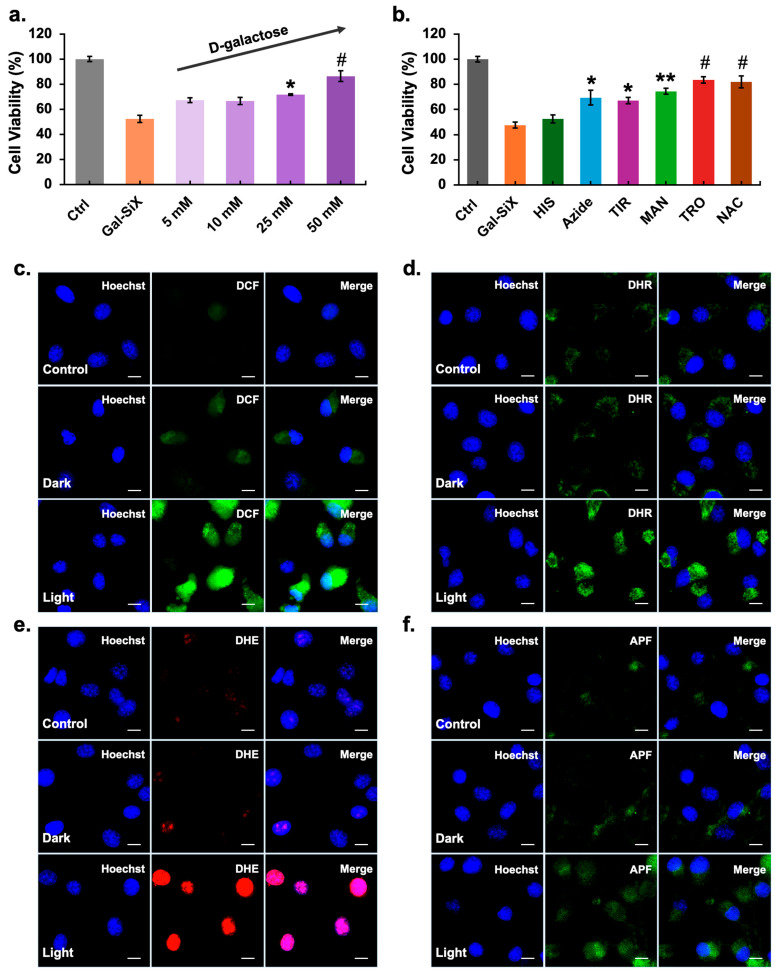
Mechanistic insight for the photodynamic application of **Gal-SiX** in U87MG cells. (**a**) Cell viability analysis after pre-treatment with increasing concentrations of D-galactose (5–50 mM, 4 h incubation), then post-treated with **Gal-SiX** (2.5 μM, 1 h incubation), followed by LED irradiation (595 nm, 8.12 mW/cm^2^, 2 h) and 24 h dark incubation (*n* = 6). (**b**) Cell viability analysis after co-treatment with various ROS scavengers, histidine (HIS, 5 mM), sodium azide (5 mM), Tiron (TIR, 100 µM), mannitol (MAN, 25 mM), Trolox (TRO, 25 µM), and N-acetylcysteine (NAC, 5 mM) prior to **Gal-SiX** treatment and PDT (*n* = 6). Intracellular ROS detection after PDT application with **Gal-SiX** using different fluorescent probes: (**c**) DCF (Green) for general ROS, (**d**) DHR123 (Green) for hydrogen peroxide and mitochondrial ROS, (**e**) DHE (Red) for superoxide anion, and (**f**) APF (Green) for hydroxyl radical and peroxynitrite. Control, dark (without irradiation), and light (irradiated) conditions are presented for each probe. Nuclei stained with Hoechst 33,342 (blue). Scale bars: 10 μm. * *p* < 0.05, ** *p* < 0.01, # *p* < 0.001 vs. untreated cells.

Notably, a strong intracellular signal was observed after 1 h, which further intensified at 2 and 4 h. In contrast, L929 cells displayed comparatively lower fluorescence intensity at all time points, suggesting reduced uptake and/or activation efficiency in non-tumor cells. These findings confirm selective and gradual intracellular accumulation of **Gal-SiX** in glioblastoma cells, supporting its tumor-targeted activation profile after 1 h of incubation (Figure 3c). This supports the statistically significant phototoxicity profile of **Gal-SiX** in U87MG cells compared to L929 cells (Figure 3d). To further support the *β*-galactosidase-dependent activation of **Gal-SiX**, U87MG cells were pre-incubated with increasing concentrations of D-galactose (5–50 mM) as a competitive inhibitor [94]. A concentration-dependent increase in cell viability was observed with D-galactose pre-treatment, confirming that **Gal-SiX** activation is specifically mediated by endogenous *β*-galactosidase activity (Figure 4a).

Following confirmation of cellular uptake and activation, the underlying mechanism of cell death was elucidated. For this purpose, apoptotic markers were assessed. Live/dead cell staining using acridine orange and ethidium bromide (EtBr) revealed a substantial increase in red fluorescence in U87MG cells at IC_50_, consistent with extensive loss of plasma membrane integrity and chromatin condensation, indicative of late apoptosis and secondary necrosis [95,96,97]. The predominance of EtBr-positive nuclei and fragmented or swollen morphologies supports activation of terminal cell death programs rather than transient stress responses [97,98,99]. In contrast, L929 cells showed predominantly green fluorescence with intact nuclear morphology, suggesting higher viability and minimal induction of apoptosis or necrosis (Figure 3e).

#### 3.3.3. Subcellular Localization

Mitochondria are critical regulators of intrinsic apoptosis, as their photodamage can lead to mitochondrial membrane depolarization, cytochrome c release, and activation of downstream pathways. Similarly, lysosomal targeting in PDT has been shown to induce lysosomal membrane permeabilization, resulting in the release of cathepsins and subsequent activation of apoptotic or necrotic pathways. The preferential accumulation of photosensitizers in mitochondria and lysosomes is therefore considered a critical determinant of PDT efficacy, as both organelles are highly susceptible to localized oxidative stress and serve as irreversible checkpoints for intracellular damage [100].

To investigate the intracellular trafficking and to determine the subcellular localization of **Gal-SiX**, U87MG cells were stained with organelle-specific trackers (Figure S9). Confocal images demonstrated that **Gal-SiX** predominantly accumulates in mitochondria and lysosomes, as evidenced by substantial overlap with mitotracker (PCC = 0.79 ± 0.02) and lysotracker (PCC = 0.82 ± 0.03) (Figure 3f), while partial distribution was observed in the endoplasmic reticulum (PCC = 0.60 ± 0.04) [101]. We believe that the accumulation of **Gal-SiX** in mitochondria and lysosomes plays a key role in mediating its phototoxic effects in glioblastoma cells.

#### 3.3.4. Mechanistic Insights into Type I/II Mediated Phototoxicity

To determine whether the observed cytotoxicity resulted from **Gal-SiX**-induced oxidative stress, intracellular ROS generation was examined in U87MG cells. Robust green fluorescence was observed with DCF staining (general ROS; Figure 4c) and DHR123 (mitochondrial ROS; Figure 4d) after LED irradiation, highlighting extensive intracellular oxidative stress. Notably, strong red fluorescence from DHE (superoxide anion; Figure 4e) and partial green fluorescence from APF (hydroxyl radical and peroxynitrite; Figure 4f) were evident under light-activated conditions, supporting predominant Type I ROS production. In contrast, minimal fluorescence emission was observed in dark or control groups, confirming the strict light-dependent activation of **Gal-SiX**.

Moreover, based on the data obtained from cell viability assays in the presence of scavengers (Figure 4b), co-treatment with tiron (superoxide anion scavenger), mannitol (hydroxyl radical scavenger), trolox (peroxyl radical scavenger), and N-acetylcysteine (broad-spectrum thiol-based ROS scavenger) significantly rescued cell viability following photodynamic application, indicating a substantial contribution of Type I ROS pathways. A substantial but relatively lower protective effect was also observed with sodium azide and, to a lesser extent, with histidine, which primarily quench singlet oxygen (^1^O_2_), suggesting that Type II mechanisms also play an essential role in **Gal-SiX**-mediated phototoxicity, but to a lesser extent than Type I. Type I-biased systems show predominant inhibition of phototoxicity upon radical scavenging, consistent with your interpretation that superoxide, hydroxyl and peroxyl radicals substantially contribute to cell death [102,103]. Additionally, the effects of ROS scavengers on intracellular DCF fluorescence were further visualized using confocal microscopy and are presented in Supplementary Figure S10, supporting the viability assay results.

Collectively, the results demonstrate that **Gal-SiX** acts as a dual Type I/II agent, generating superoxide anions, hydroxyl radicals, peroxynitrite, and singlet oxygen in tumor cells, thereby inducing effective, enzyme-activated phototoxicity. This dual effect of **Gal-SiX** makes it advantageous for overcoming hypoxic tumor microenvironments where pure Type II agents are often less efficient. These findings align well with the broader literature showing that dual Type I/II photosensitizers can generate both radical species (superoxide, hydroxyl radical, peroxynitrite-derived chemistry) and singlet oxygen in tumor cells, resulting in potent and often hypoxia-tolerant phototoxicity [104]. Reviews and mechanistic studies further emphasize that such dual-mode PSs are particularly advantageous in hypoxic tumor microenvironments, where purely Type II (singlet oxygen-only) agents lose efficiency, whereas Type I radical pathways remain effective and can even be enhanced, leading to more reliable and selective PDT outcomes in solid tumors [105,106].

#### 3.3.5. Dual Type I/II-Based Oxidative Mechanisms Underlying Cell Death

Further analyses were conducted to elucidate the mechanistic insights underlying the cell death induced by **Gal-SiX**. Lipid peroxidation, a key endpoint of ROS-mediated membrane damage, was evaluated [93]. The results demonstrated that **Gal-SiX** induced a significant increase in lipid peroxidation immediately after light exposure (133.70 ± 2.27%) (** *p* < 0.01 vs. control), which further enhanced during the post-irradiation period and reached drastic levels at 24 h (304.67 ± 2.92%) (# *p* < 0.001 vs. control) in U87MG cells (Figure S11a). This was further supported by the sulfo-phospho-vanillin assay [94], which confirmed a pronounced elevation of lipid products in **Gal-SiX**-treated cells. A marked accumulation of free lipid content was detected at 24 h under LED irradiation (206.19 ± 14.43%) (# *p* < 0.001 vs. control), consistent with the release of unsaturated lipid products upon disruption of membranous structures such as mitochondria, lysosomes, and the plasma membrane (Figure S11b). Concomitantly, a notable depletion of intracellular thiol levels was observed after LED exposure at 24 h (80.8 ± 2.6%) (* *p* < 0.05 vs. control), indicating a depletion of glutathione-dependent antioxidant defenses and reinforcing the role of oxidative stress in the phototoxic response (Figure S11c) [90]. Taken together, increased lipid peroxidation, elevated lipid accumulation, and thiol depletion indicate that **Gal-SiX**-induced phototoxicity may involve ferroptosis-like oxidative cell death mechanisms, in addition to direct ROS-mediated membrane injury [107].

## 4. Conclusions

In summary, the first *β*-Galactosidase-activatable Si-Xanthene-based PS, **Gal-SiX**, was developed via an improved 10-step synthetic process. This PS demonstrated a remarkable turn-on response in both absorption and fluorescence signals, with a significant capacity to generate ROS in an aqueous medium upon activation by β-galactosidase. The potential of **Gal-SiX** as an activatable PS was then tested in vitro on glioblastoma (U87MG) and healthy (L929) cell lines, where it showed no dark toxicity toward either cell type. Upon irradiation, the presence of the *β*-Gal moiety enabled light-induced cytotoxicity of glioblastoma cells (IC_50_ values of 3.30 μM vs. 7.19 μM for L929). Detailed MTT and confocal microscopy analyses revealed that **Gal-SiX** exhibits a dual Type I/II mechanism of action in PDT, which is beneficial for treating hypoxic tumors. Further biochemical assays confirmed this dual mechanism, as **Gal-SiX** induced a marked increase in lipid peroxidation, accompanied by a significant accumulation of lipid degradation products and a depletion of intracellular thiols. These hallmarks of oxidative membrane damage indicate exhaustion of glutathione-dependent defenses, engagement of ferroptosis-like death pathways, and ROS-induced disruption of membranous structures. Our work on developing Type I mechanism-biased PDT agents with NIR absorption and evaluating them in hypoxic tumors with different non-apoptotic cell death mechanisms is ongoing.

## Figures and Tables

**Table 1:** Photophysical properties of **Gal-SiX** and **SiX**.

PS	*λ_abs_* (nm) ^1^	*λ_ems_* (nm) ^1^	*φ_F_* (%) ^1,2^	*Φ*_Δ_ (%) ^1,3^
**Gal-SiX**	486	Not detectable	Not detectable	n.d. ^d^
**SiX**	598	613	6.4	52

^1^ In PBS (pH 7.4, 1% DMSO. ^2^ Reference: Tokyo Magenta in PBS (***φ_F_*** = 42%) [54]. ^3^ Reference: methylene blue in PBS (Φ_Δ_ = 52%) [79], ^d^ Not determined.

## Data Availability

The raw data supporting the conclusions of this article will be made available by the authors on request.

## Supplementary Materials

The following supporting information can be downloaded at https://www.mdpi.com/article/10.3390/pharmaceutics18040420/s1. Figure S1. HPLC spectrum of Gal-SiX. Figure S2. Absorbance profile of time dependent activation of Gal-SiX (10 μM) in PBS buffer (pH 7.4, 1% DMSO) upon incubation with β-galactosidase (5 U). Figure S3. (a) Absorbance spectrum of Gal-SiX (20 μM) in cell culture medium up to 1 h dark incubation, then irradiation with a 595 nm LED light source (3.50 mW/cm^2^) for up to 2 h. (b) Time-dependent decrease in absorbance of Gal-SiX (20 μM) (Dark 60, LED 15, LED 30, LED 60, LED 120). (c) Absorbance spectra of SiX (20 μM) in cell culture medium up to 1 h dark incubation with β-galactosidase (5 U), then irradiation with a 595 nm LED light source (at 10 cm distance, 3.50 mW/cm^2^) for up to 2 h. (Irradiation was performed from a distance of 10 cm.) (d) Time-dependent decrease in absorbance of SiX (20 μM) (Dark 60, LED 15, LED 30, LED 60, LED 120). Figure S4. Reaction between photosensitized 1O2 and ADMDA. Figure S5. Decrease in the absorption signal of ADMDA in PBS (pH 7.4, 1% DMSO) upon irradiation of Gal-SiX (10 μM, at 10 cm distance, 3.50 mW/cm^2^) at 380 nm with respect to time. Figure S6. Absorbance spectrum of Gal-SiX (10 μM) in PBS buffer (pH 7.4, 1% DMSO) upon treatment with β-galactosidase and various interferents (orange), or in the absence of β-galactosidase with the same interferents (blue). Figure S7. Cell viabilities of U87MG (a–d) and L929 (e,f) treated with the increasing concentrations of Gal-SiX for 24 h (a,e) or 0.5, 1 and 2 h at dark, followed by 0.5 h (b,f), 1 h (c,g) or 2 h (d,h) LED (595 nm, 8.12 mW/cm^2^) exposure and subsequent incubation in the dark for 24 h. (*n* = 6–8). Figure S8. Time-dependent activation and internalization of Gal-SiX (1 µM) for 0.5, 1, 2, and 4 h in U87MG and L929 cells. Blue, Hoechst 33342; Red, Gal-SiX. Scale bar: 10 μm. Figure S9. Subcellular localization of Gal-SiX (2.5 µM) in U87MG cells visualized by confocal microscopy after 1-h incubation (a). Scatter plot graphs for Mitotracker and Lysotracker mentioned in Figure 3f (b). Blue: Hoechst 33342 (nuclei); Red: Gal-SiX; Green: ER-Tracker™ Green (ER), MitoTracker™ Green FM (mitochondria), or LysoTracker™ Yellow HCK-123 (lysosomes). Scale bar: 10 µm. Figure S10. Representative confocal images for intracellular ROS generation in U87MG cells treated with Gal-SiX (2.5 μM, 1h), followed by LED irradiation for 2 h with/without ROS scavengers. ROS scavengers: Tiron (TIR, 100 μM) for superoxide anion, mannitol (MAN, 25 mM) for hydroxyl radical, histidine (HIS, 5 mM) and sodium azide (NaN_3_, 5 mM) for singlet oxygen, Trolox (TRO, 25 μM) for peroxyl radicals, and N-acetylcysteine (NAC, 5 mM) as a general ROS scavenger. Blue: Hoechst 33342 (nuclei); Green: DCF. Scale bar: 20 μm. Figure S11. Effects of Gal-SiX on oxidative stress–related parameters in U87MG cells under dark and LED (595 nm, 8.12 mW/cm^2^) irradiation conditions. (a) Lipid peroxidation levels (%) assessed at the end of treatment (Time D) and 24 h post-irradiation (Post 24). (b) Lipid content (%) measured at Time D and Post 24. A pronounced increase in lipid accumulation was observed in Gal-SiX–treated cells under LED exposure at 24 h. (c) Thiol content (%) determined at Time D and Post 24. A significant reduction in cellular thiol levels was detected in Gal-SiX–treated cells after LED irradiation at 24 h. * *p* < 0.05, ** *p* < 0.01 vs. control at corresponding conditions; # *p* < 0.001 vs. post-24 h control. (*n* = 3–4). Figure S12. 1H NMR spectrum of compound **1** in CDCl3. Figure S13. 13C NMR spectrum of compound **1** in CDCl3. Figure S14. 1H NMR spectrum of compound **2** in CDCl3. Figure S15. 13C NMR spectrum of compound **2** in CDCl3. Figure S16. 1H NMR spectrum of compound **3** in CDCl3. Figure S17. 13C NMR spectrum of compound **3** in CDCl3. Figure S18. 1H NMR spectrum of compound **4** in MeOD. Figure S19. 13C NMR spectrum of compound **4** in MeOD. Figure S20. 1H NMR spectrum of compound **5** in MeOD. Figure S21. 13C NMR spectrum of compound **5** in MeOD. Figure S22. 1H NMR spectrum of compound **6** in CDCl3. Figure S23. 13C NMR spectrum of compound **6** in CDCl3. Figure S24. 1H NMR spectrum of compound **7** in CDCl3. Figure S25. 13C NMR spectrum of compound **7** in CDCl3. Figure S26. 1H NMR spectrum of compound **8** in CDCl3. Figure S27. 13C NMR spectrum of compound **8** in CDCl3. Figure S28. 1H NMR spectrum of compound **9** in CDCl3. Figure S29. 13C NMR spectrum of compound **9** in CDCl3. Figure S30. 1H NMR spectrum of Gal-SiX in MeOD. Figure S31. 13C NMR spectrum of Gal-SiX in MeOD. Figure S32. HRMS of **SiX.** Table S1. HPLC retention times and peak areas of **Gal-SiX**. Table S2. The IC_50_ values (µM) of **Gal-SiX** in U87MG and L929 cells under various treatment and LED (595 nm, 8.12 mW/cm^2^) irradiation conditions. Table S3. The phototoxicity profile of **Gal-SiX**.

## Abbreviations

The following abbreviations are used in this manuscript:

ADMDA	2,2′-(Anthracene-9,10-diyl)bis(methylene)dimalonic acid
AO	Acridine orange
APF	2-[6-(4′-Amino)phenoxy-3H-xanthen-3-on-9-yl]benzoic acid
BBB	Blood–brain barrier
BCA	Bicinchoninic acid
CLSM	Confocal laser scanning microscopy
DCF	2′,7′-Dichlorofluorescein
DCFH-DA	2′,7′-Dichlorofluorescin diacetate
DCM	Dichloromethane
DHE	Dihydroethidium
DHR123	Dihydrorhodamine 123
DMEM	Dulbecco’s modified Eagle medium
DMF	Dimethylformamide
DTNB	5,5′-Dithiobis(2-nitrobenzoic acid)
Eq.	Equivalent
EtBr	Ethidium bromide
EtOAc	Ethyl acetate
FBS	Fetal bovine serum
FDA	U.S. Food and Drug Administration
ΦΔ	Singlet oxygen quantum yield
ΦF	Fluorescence quantum yield
GBM	Glioblastoma multiforme
HIF-1α	Hypoxia-inducible factor 1 alpha
HPLC	High-performance liquid chromatography
HRMS	High-resolution mass spectrometry
IC_50_	Half-maximal inhibitory concentration
ISC	Intersystem crossing
LED	Light-emitting diode
MeCN	Acetonitrile
MeOH	Methanol
MTT	3-(4,5-Dimethylthiazol-2-yl)-2,5-diphenyltetrazolium bromide
NIR	Near-infrared
NMR	Nuclear magnetic resonance
^1^O_2_	Singlet oxygen
ONOO^−^	Peroxynitrite
PBS	Phosphate-buffered saline
PCC	Pearson’s correlation coefficient
Pd–C	Palladium on carbon
PDT	Photodynamic therapy
PI	Phototoxicity index
PS	Photosensitizer
QY	Quantum yield
RIPA	Radioimmunoprecipitation assay buffer
ROS	Reactive oxygen species
SA-*β*-gal	Senescence-associated β-galactosidase
SD	Standard deviation
SiX	*β*-Galactosidase-cleaved iodinated Si-xanthene
SOSG	Singlet Oxygen Sensor Green
TBARS	Thiobarbituric acid reactive substances
THF	Tetrahydrofuran
TI	Therapeutic index
TLC	Thin-layer chromatography
TM	Tokyo Magenta
*β*-gal	*β*-Galactosidase
