# Supplementary material for: Activatable Silicon-Xanthene Photosensitizer for Photodynamic Therapy of Glioblastoma
Source: Pharmaceutics. 2026 Mar 29;18(4):420. doi: 10.3390/pharmaceutics18040420 (PMC13118389; doi:10.3390/pharmaceutics18040420)
Supplement: Supplementary file 1 [file pharmaceutics-18-00420-s001.zip › pharmaceutics-4115200-supplementary.pdf]

Supporting Information

for

Activatable Silicon-Xanthene Photosensitizer for Photodynamic Therapy of Glioblastoma

Osman Karaman [1,†], Dilay Kepil [1,†], Mehrdad Forough [1,‡], Zubeyir Elmazoglu [1,2,*] and Gorkem Gunbas [1,*]

1 Department of Chemistry, Middle East Technical University, 06800 Ankara, Türkiye; karaman@metu.edu.tr (O.K.); dilay.kepil@metu.edu.tr (D.K.); mehrdadforogh@gmail.com (M.F.)
2 Department of Pharmacology, Ankara Medipol University, 06050 Ankara, Türkiye
* Correspondence: zubeyir.elmazoglu@ankaramedipol.edu.tr (Z.E.); ggunbas@metu.edu.tr (G.G.)
† These authors contributed equally to this work.
‡ Current address: R&D and Innovation Department, Nanografi Nanotechnology AŞ, METU Technopolis, 06531 Ankara, Türkiye.

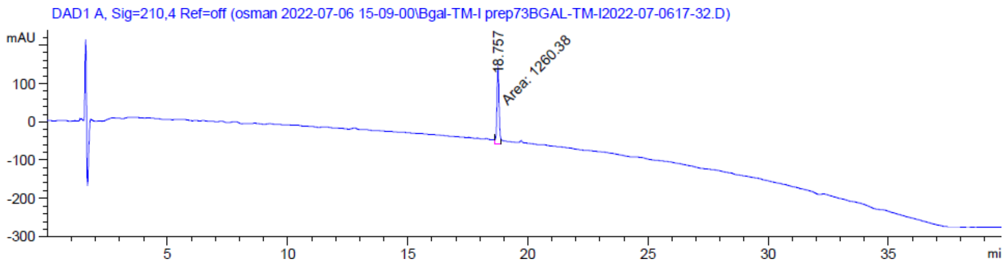

Figure S1. HPLC spectrum of **Gal-SiX**.

Table S1. HPLC retention times and peak areas of **Gal-SiX**.

Peak	Ret. Time (min)	Area (%)
**1**	18.757	100.0

Photophysical Characterization

Enzymatic Activation

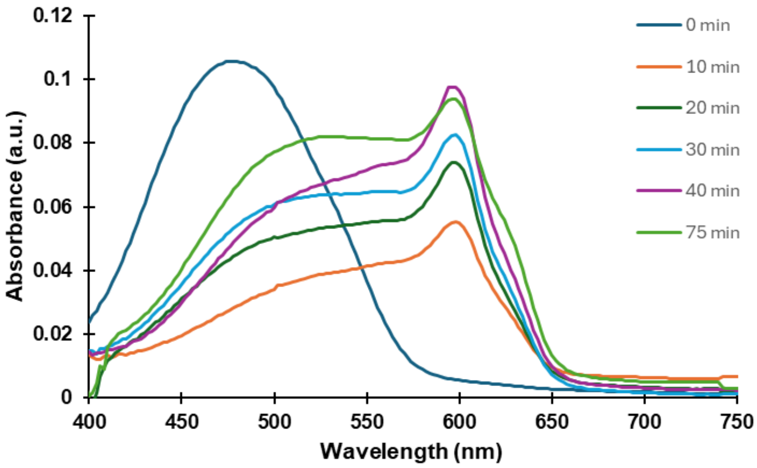

Figure S2. Absorbance profile of time dependent activation of **Gal-SiX** (10 μM) in PBS buffer (pH 7.4, 1% DMSO) upon incubation with β-galactosidase (5 U)

Photostability

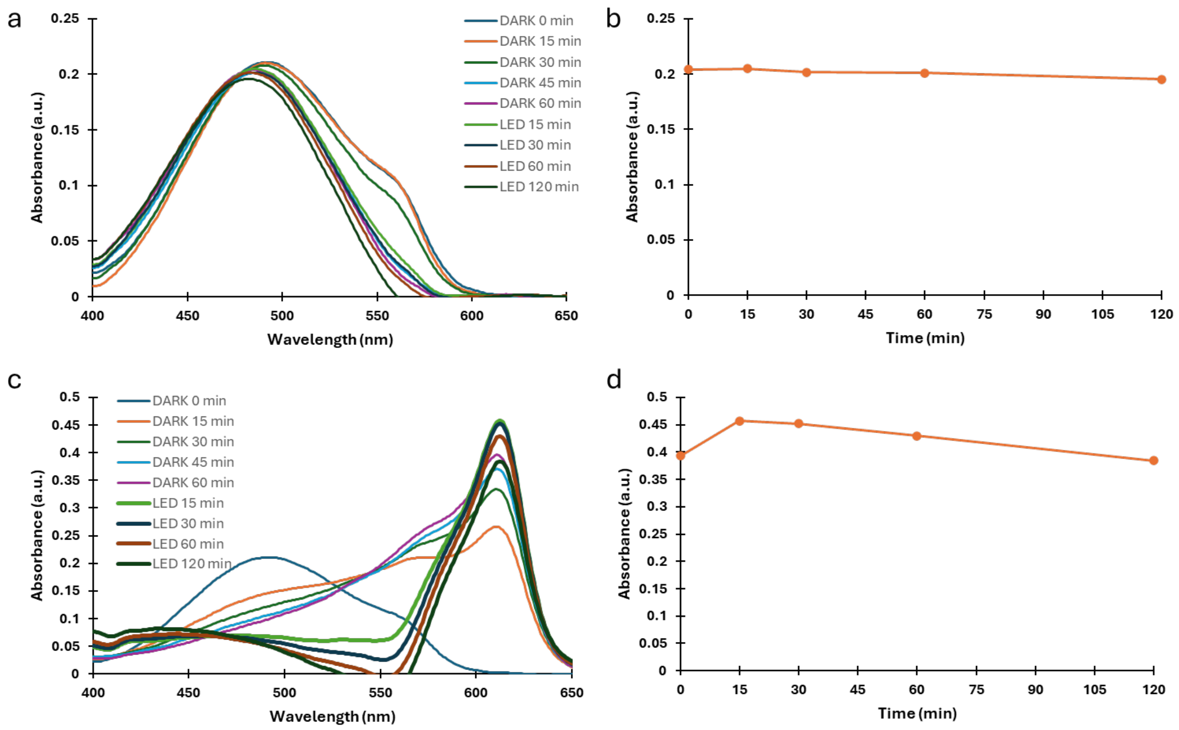

Figure S3 a. Absorbance spectrum of ***Gal-SiX*** (20 μM) in cell culture medium up to 1 h dark incubation, then irradiation with a 595 nm LED light source (3.50 mW/cm^2) for up to 2 h. **b.** Time-dependent decrease in absorbance of ***Gal-SiX*** (20 μM) (Dark 60, LED 15, LED 30, LED 60, LED 120). **c.** Absorbance spectra of **SiX** (20 μM) in cell culture medium up to 1 h dark incubation with β-galactosidase (5 U), then irradiation with a 595 nm LED light source (at 10 cm distance, 3.50 mW/cm^2) for up to 2 h. (Irradiation was performed from a distance of 10 cm.) d. Time-dependent decrease in absorbance of **SiX** (20 μM) (Dark 60, LED 15, LED 30, LED 60, LED 120).

Singlet Oxygen Trap Experiment

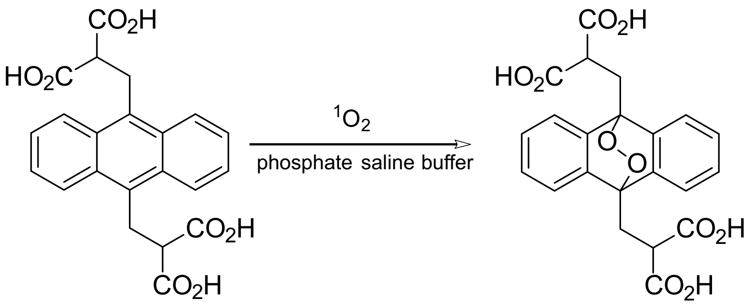

Figure S4. Reaction between photosensitized $^1O_2$ and ADMDA.

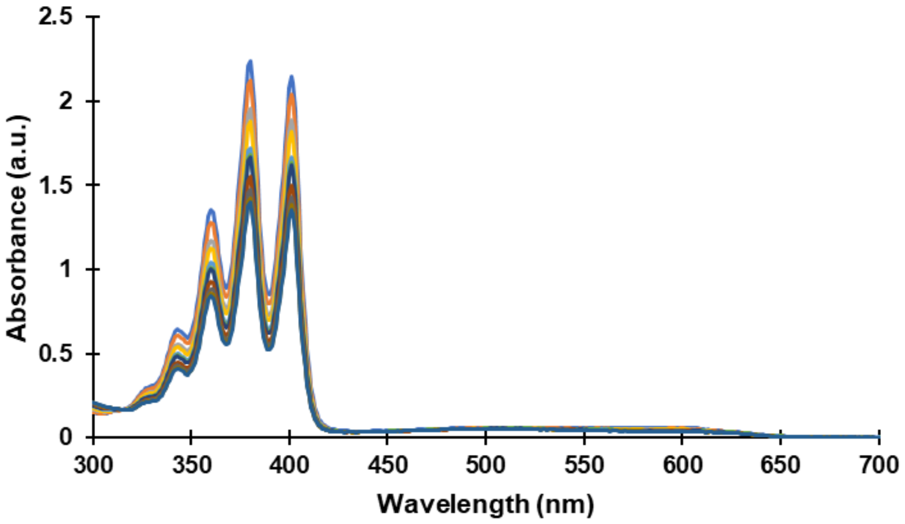

Figure S5. Decrease in the absorption signal of ADMDA in PBS (pH 7.4, 1% DMSO) upon irradiation of **Gal-SiX** (10 μM, at 10 cm distance, 3.50 mW/cm^2) at 380 nm with respect to time.

General Procedure for Detection of ROS Type

Interference Studies

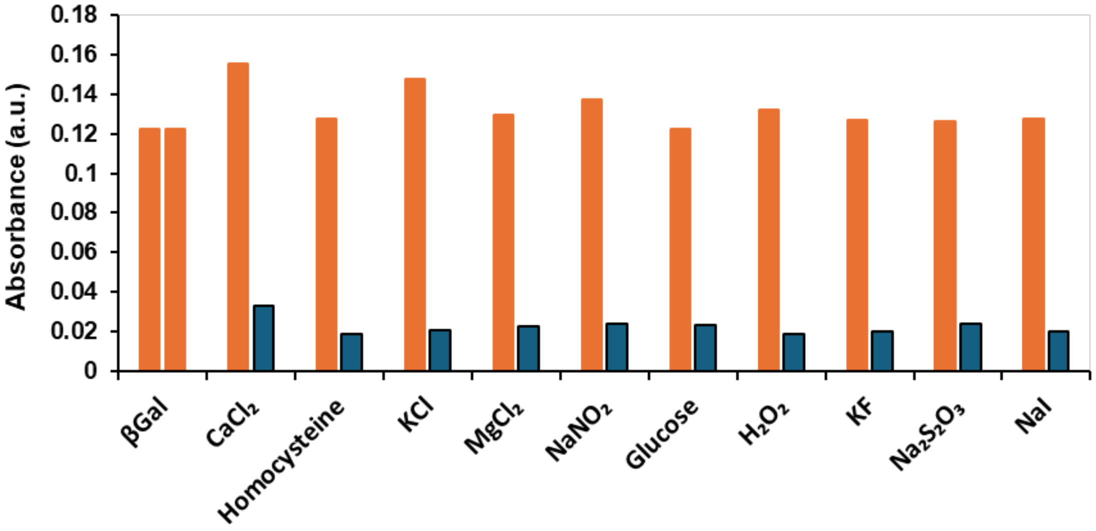

Figure S6. Absorbance spectrum of **Gal-SiX** (10 μM) in PBS buffer (pH 7.4, 1% DMSO) upon treatment with β-galactosidase and various interferents (orange), or in the absence of β-galactosidase with the same interferents (blue).

In vitro Experiments

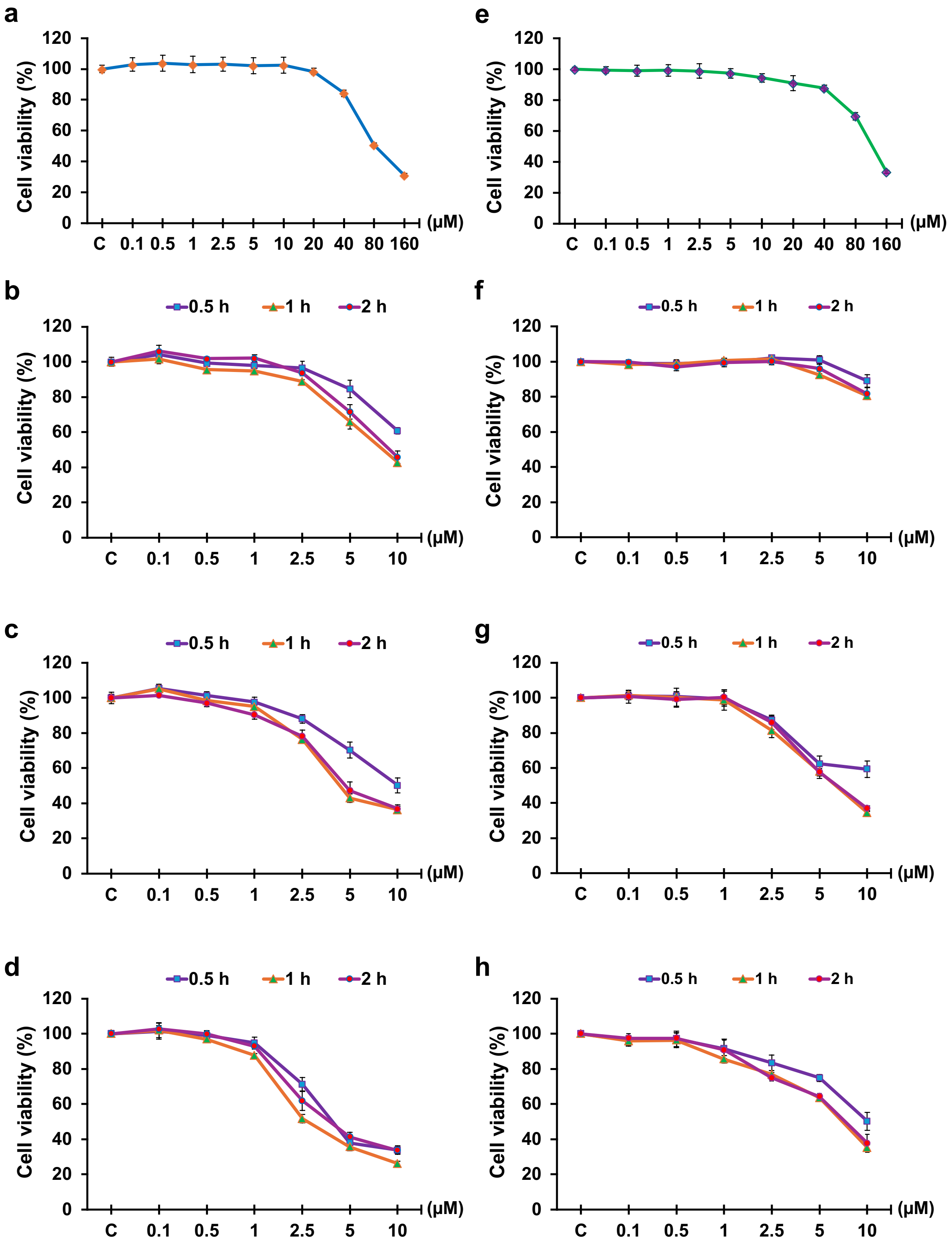

Figure S7 Cell viabilities of U87MG **(a-d)** and L929 **(e-f)** treated with the increasing concentrations of ***Gal-SiX*** for 24 h **(a,e)** or 0.5, 1 and 2 h at dark, followed by 0.5 h **(b, f)**, 1 h **(c, g)** or 2 h **(d, h)** LED (595 nm, 8.12 mW/cm^2) exposure and subsequent incubation in the dark for 24 hours. (n=6-8)

Table S2. The IC_{50} values (μM) of **Gal-SiX** in U87MG and L929 cells under various treatment and LED (595 nm, 8.12 mW/cm²) irradiation conditions

Cell Line	LED Treat	0.5 h	1 h	2 h
U87MG	0.5 h	>10	9.75±0.43	4.66±0.21
	1 h	8.16±0.22	5.312±0.25	3.30±0.11
	2 h	8.88± 0.31	5.71±0.23	4.31±0.17
L929	0.5 h	>10	>10	>10
	1 h	>10	8.01±0.63	7.19±0.32
	2 h	>10	8.65±0.7	7.98±0.61

Table S3. The phototoxicity profile of ***Gal-SiX***.

Cell Line	IC_{50} (Dark, μM)	IC_{50} (Light, μM)	PI[a]	SI[b]	TI[c]
**U87MG**	81.92±2.94	3.30±0.11	24.8	2.18	35.3
**L929**	116.6±3.34	7.19±0.32	16.2	NA	NA

[a] Phototoxicity index = $IC_{50,\ dark}$ /$IC_{50,\ light}$
[b] Selectivity index = $IC_{50,\ light,\ healthy\ cells}$/$IC_{50,\ light,\ cancer\ cells,}$
[c] *In vitro* Therapeutic index = $IC_{50,\ dark,\ healthy\ cells}$/$IC_{50,\ light,\ cancer\ cells}$

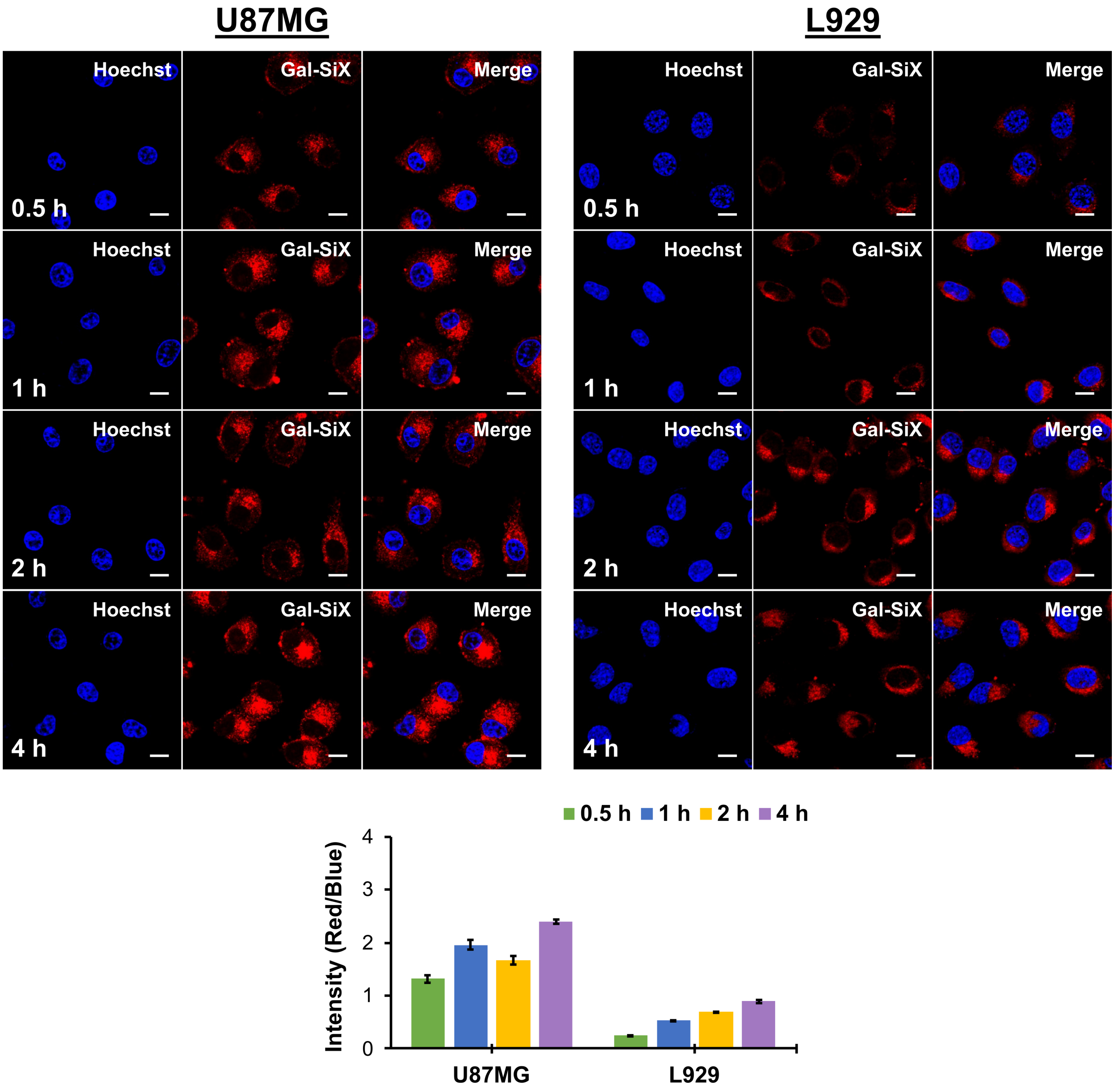

Figure S8. Time-dependent activation and internalization of **Gal-SiX** (1 μM) for 0.5, 1, 2, and 4 h in **U87MG** and **L929** cells. Blue, Hoechst 33342; Red, **Gal-SiX** . Scale bar: 10 μm.

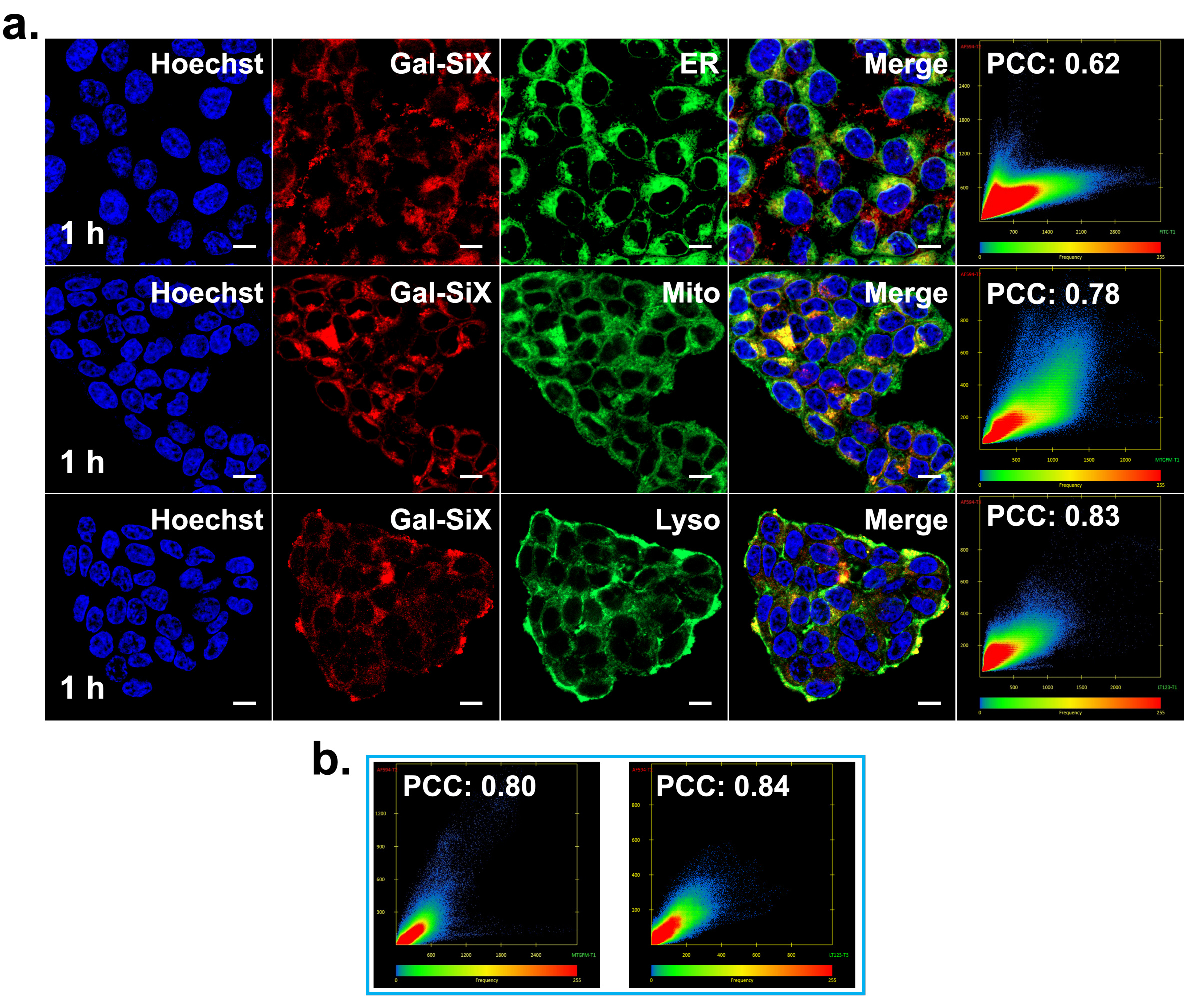

Figure S9. Subcellular localization of **Gal-SiX** (2.5 μM) in U87MG cells visualized by confocal microscopy after 1-hour incubation **(a)**. Scatter plot graphs for Mitotracker and Lysotracker mentioned in **Figure 3f (b)**. Blue: Hoechst 33342 (nuclei); Red: **Gal-SiX**; Green: ER-Tracker™ Green (ER), MitoTracker™ Green FM (mitochondria), or LysoTracker™ Yellow HCK-123 (lysosomes). Scale bar: 10 μm.

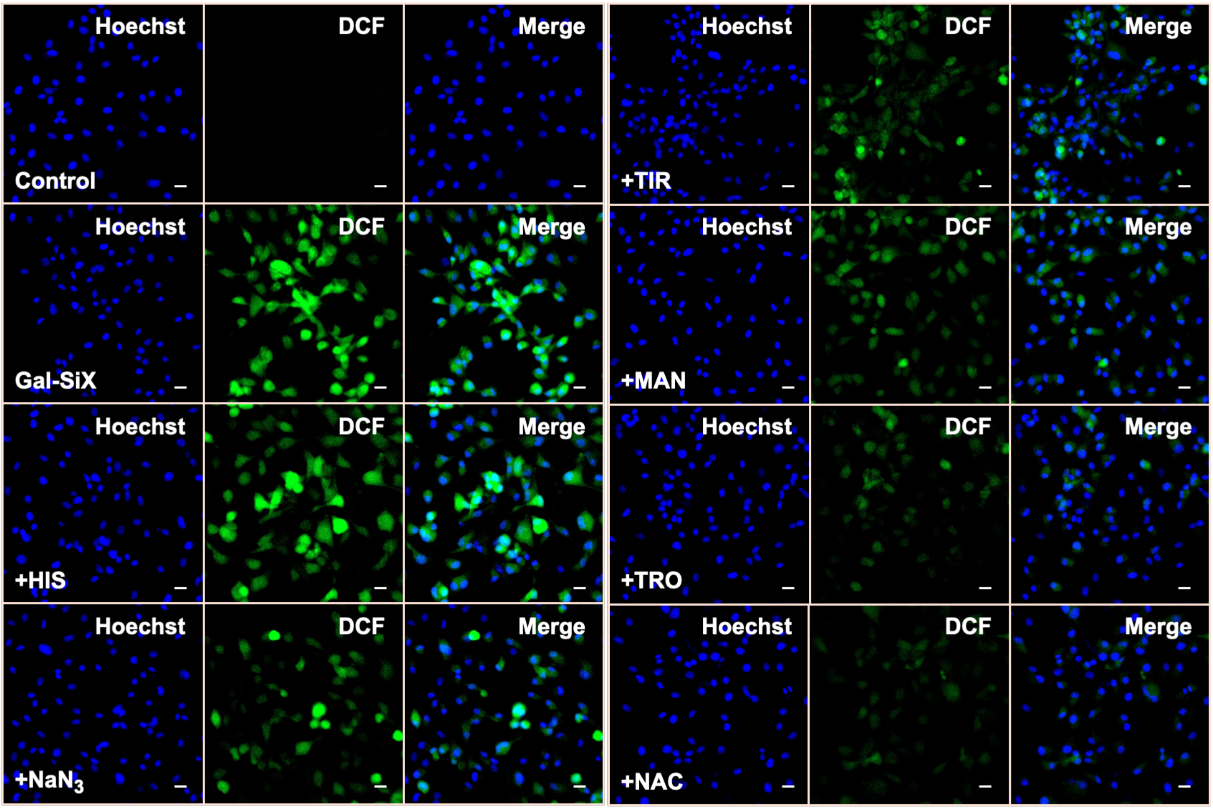

Figure S10. Representative confocal images for intracellular ROS generation in U87MG cells treated with ***Gal-SiX* (2.5 μM, 1h)**, followed by LED irradiation for 2 h with/without ROS scavengers. ROS scavengers: Tiron (TIR, 100 μM) for superoxide anion, mannitol (MAN, 25 mM) for hydroxyl radical, histidine (HIS, 5 mM) and sodium azide (NaN_3, 5 mM) for singlet oxygen, Trolox (TRO, 25 μM) for peroxyl radicals, and N-acetylcysteine (NAC, 5 mM) as a general ROS scavenger. Blue: Hoechst 33342 (nuclei); Green: DCF. Scale bar: 20 μm.

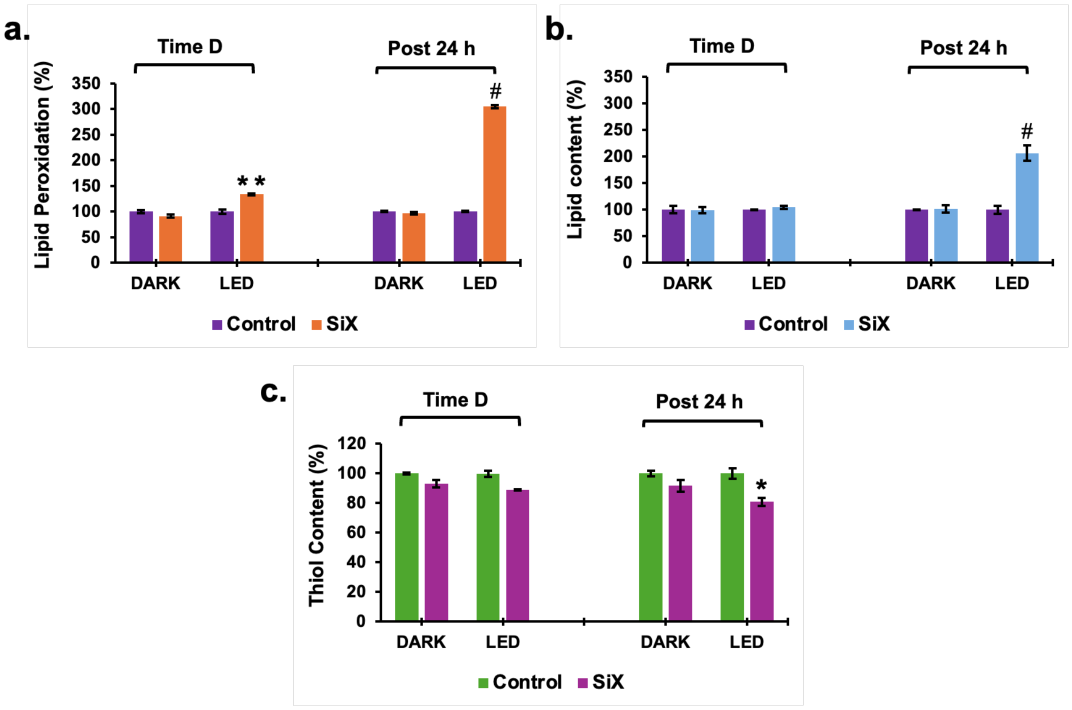

Figure S11. Effects of ***Gal-SiX*** on oxidative stress–related parameters in U87MG cells under dark and LED (595 nm, 8.12 mW/cm²) irradiation conditions. **(a)** Lipid peroxidation levels (%) assessed at the end of treatment (Time D) and 24 h post-irradiation (Post 24). **(b)** Lipid content (%) measured at Time D and Post 24. A pronounced increase in lipid accumulation was observed in ***Gal-SiX***–treated cells under LED exposure at 24 h**. (c)** Thiol content (%) determined at Time D and Post 24. A significant reduction in cellular thiol levels was detected in ***Gal-SiX***–treated cells after LED irradiation at 24 h. *p < 0.05, **p < 0.01 vs. control at corresponding conditions; #p < 0.001 vs. post-24 h control. (n=3-4)

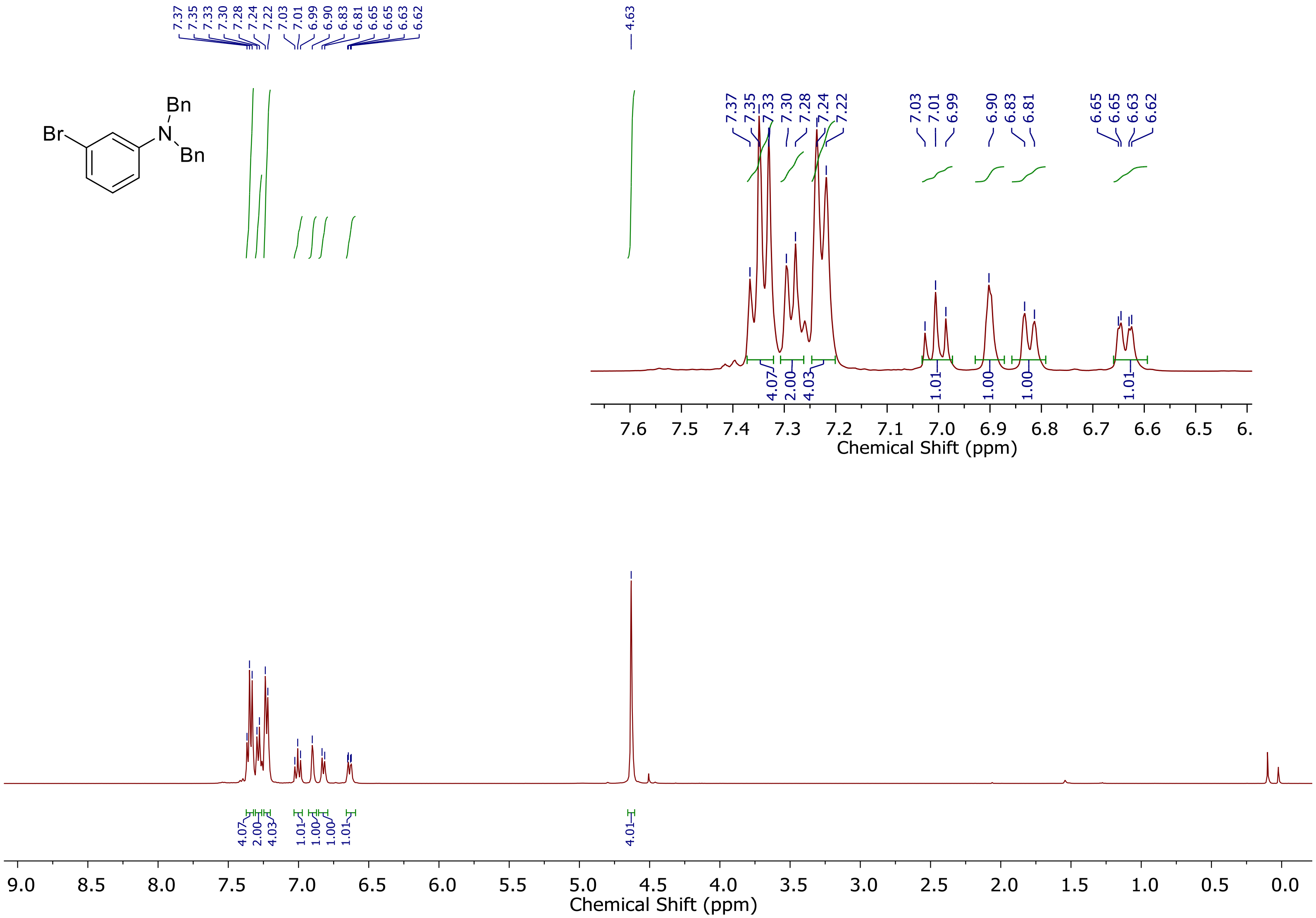

Figure S12. ^{1}H NMR spectrum of compound **1** in $CDCl_3$.

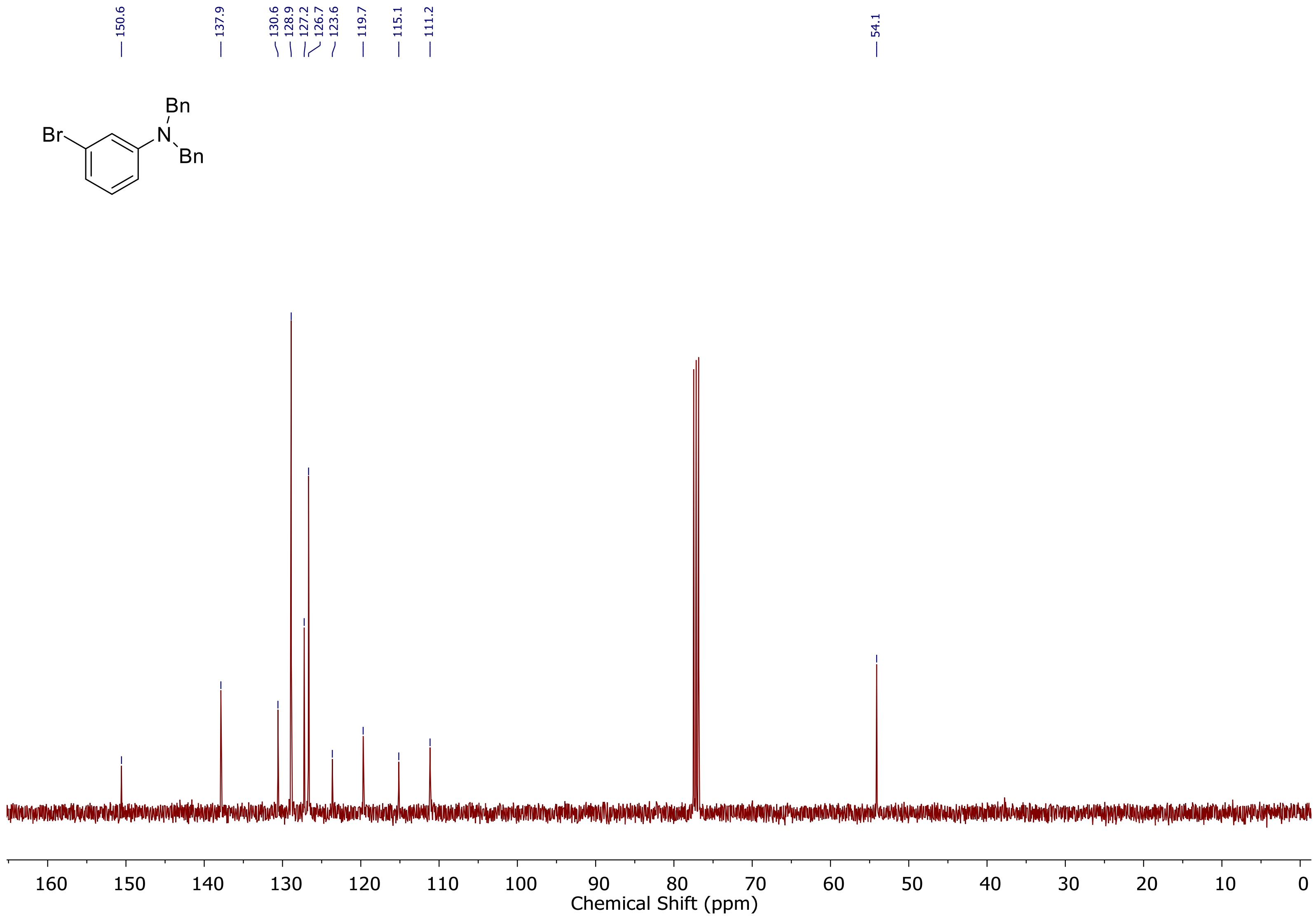

Figure S13. ^{13}C NMR spectrum of compound **1** in $CDCl_3$.

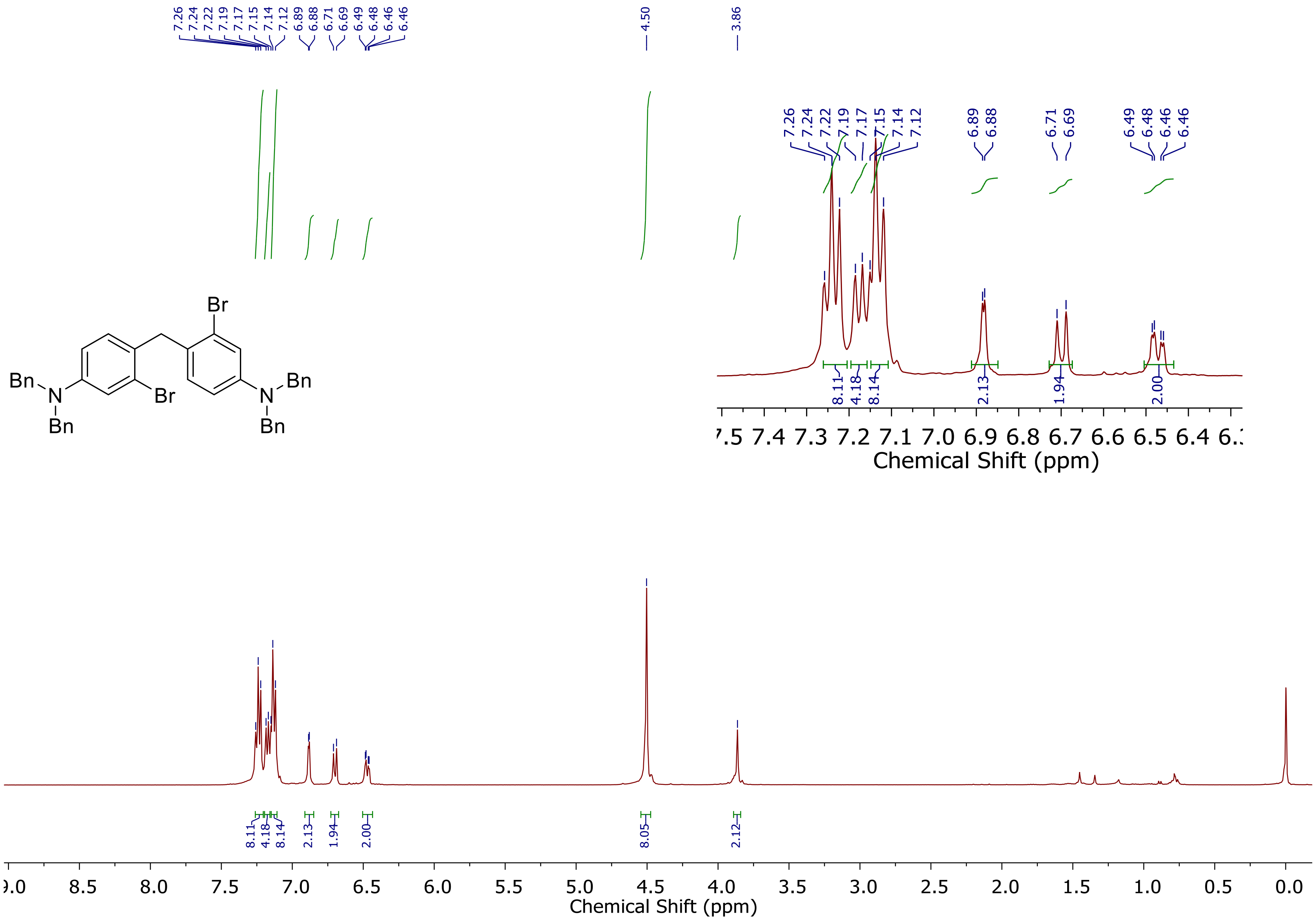

Figure S14. ^{1}H NMR spectrum of compound **2** in $CDCl_3$.

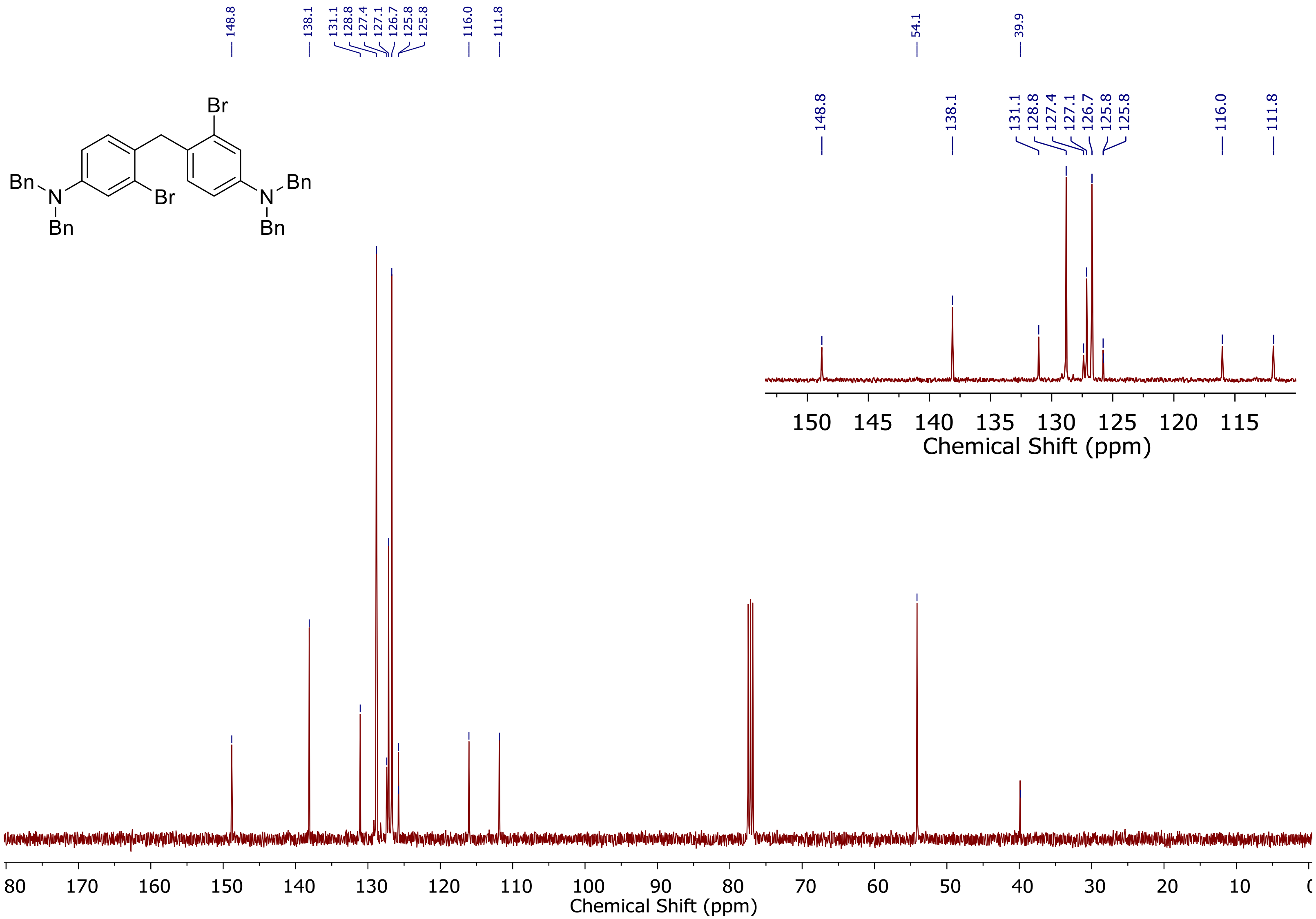

Figure S15. ^{13}C NMR spectrum of compound **2** in $CDCl_3$.

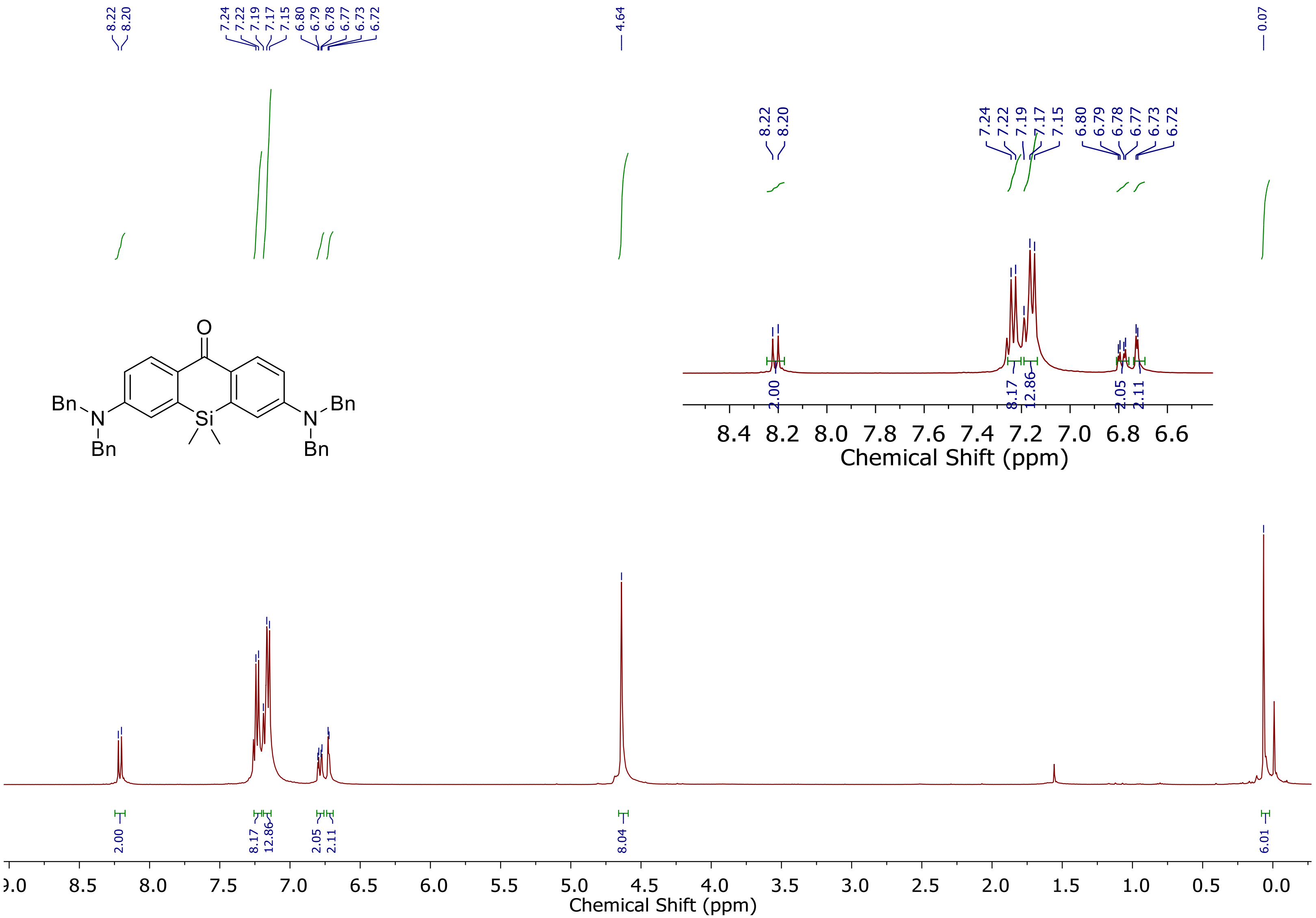

Figure S16.^{1}H NMR spectrum of compound **3** in $CDCl_3$.

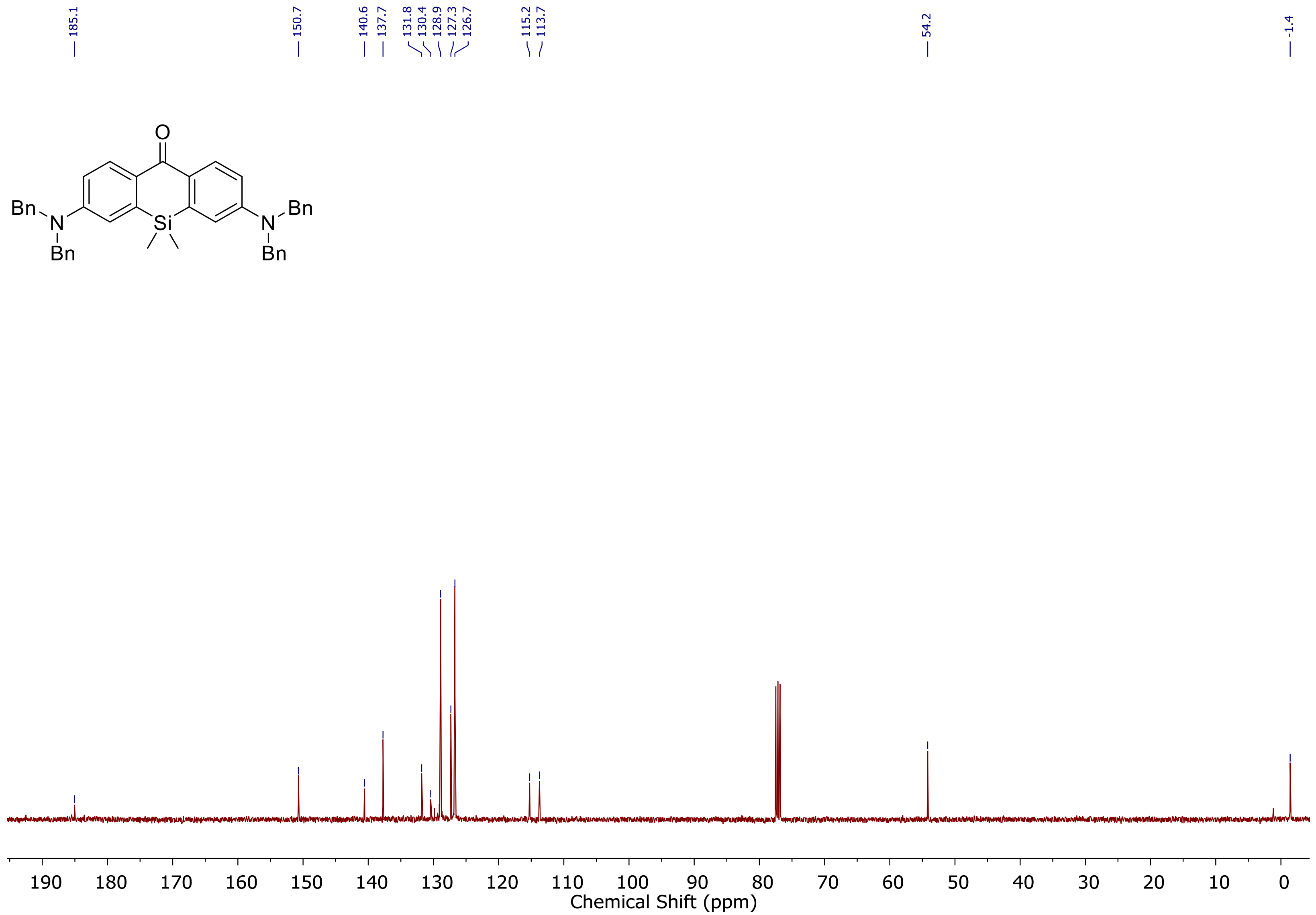

Figure S17. ^{13}C NMR spectrum of compound **3** in $CDCl_3$.

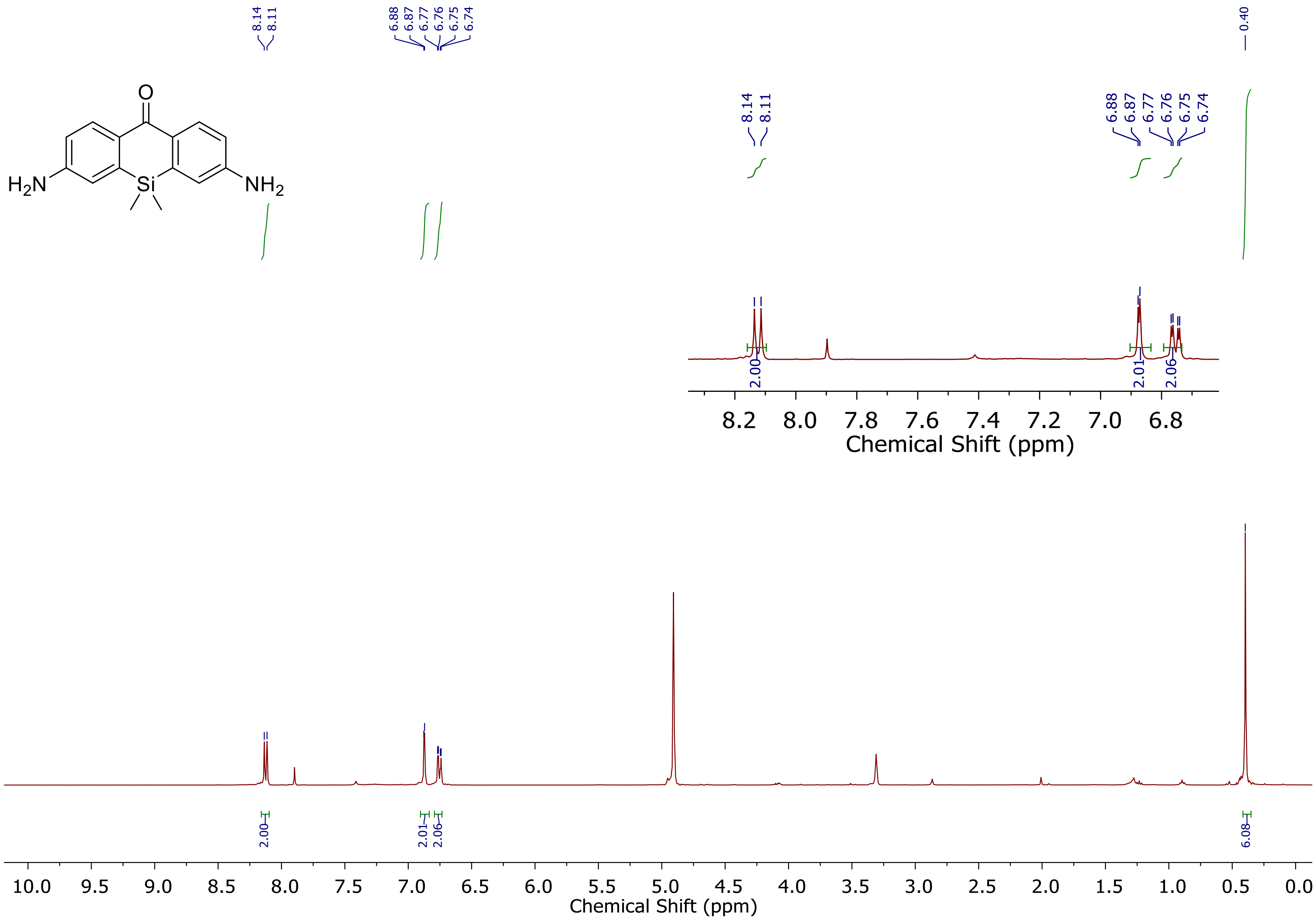

Figure S18. ^{1}H NMR spectrum of compound **4** in MeOD.

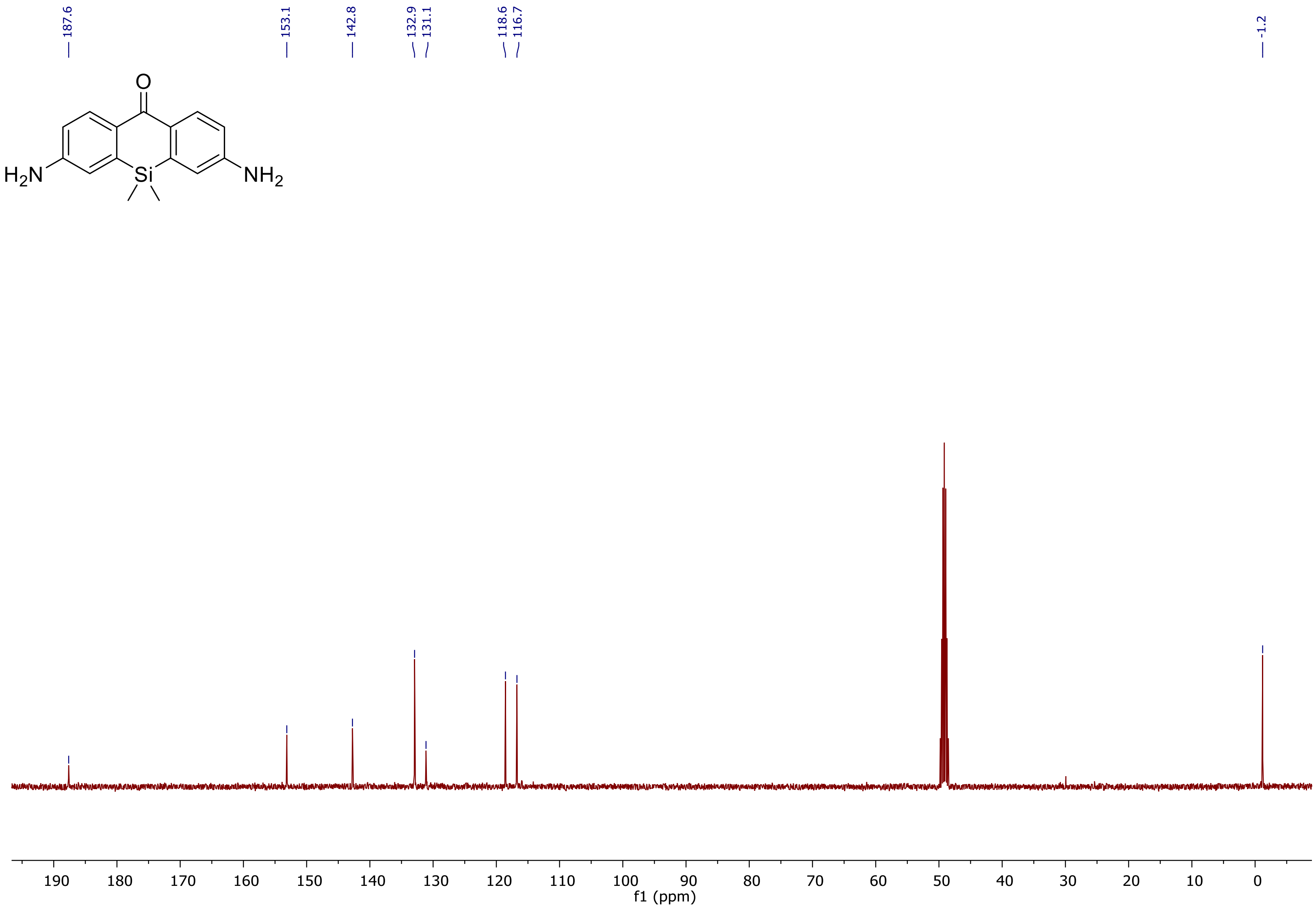

Figure S19. ^{13}C NMR spectrum of compound **4** in MeOD.

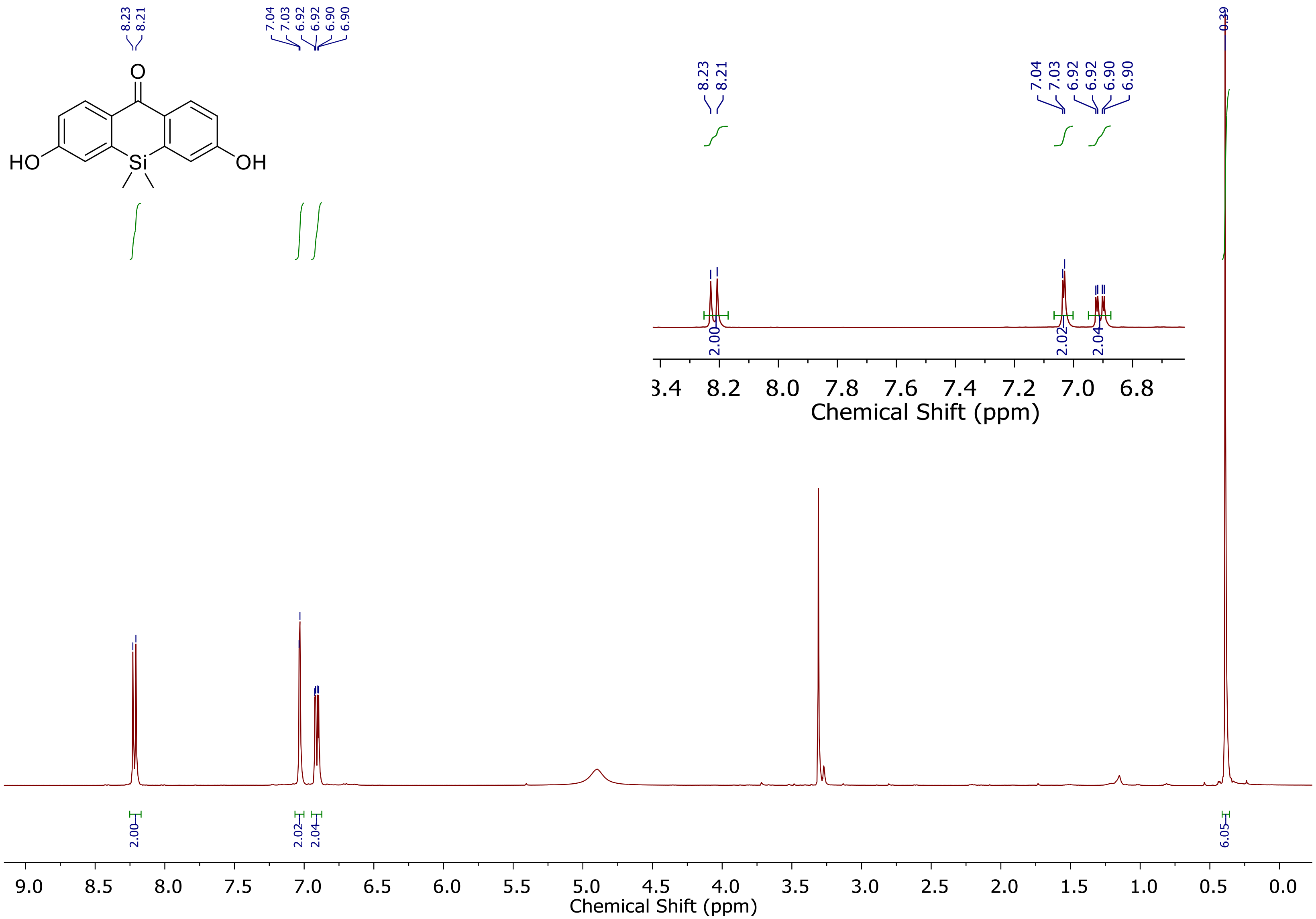

Figure S20. ^{1}H NMR spectrum of compound **5** in MeOD.

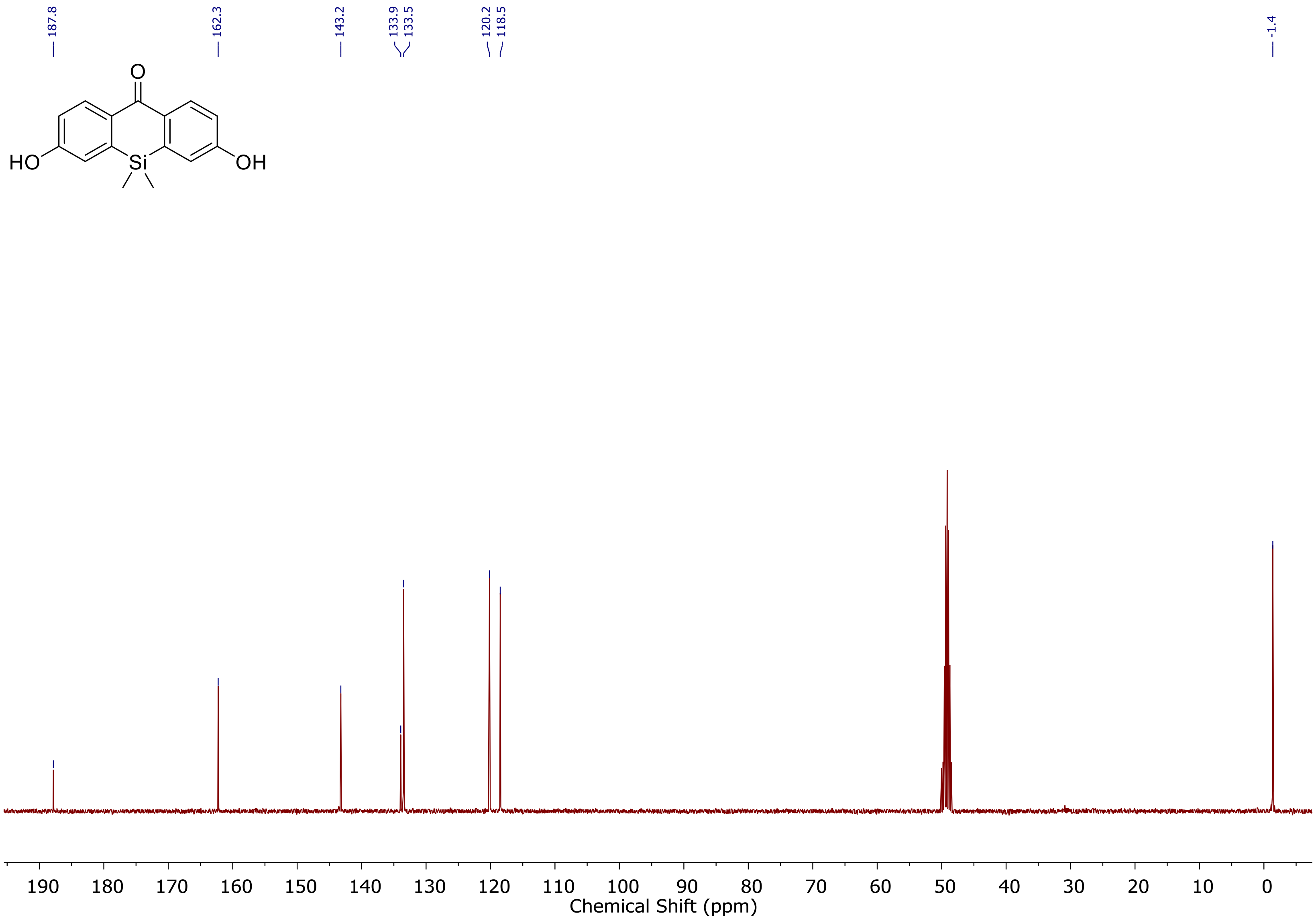

Figure S21. ^{13}C NMR spectrum of compound **5** in MeOD.

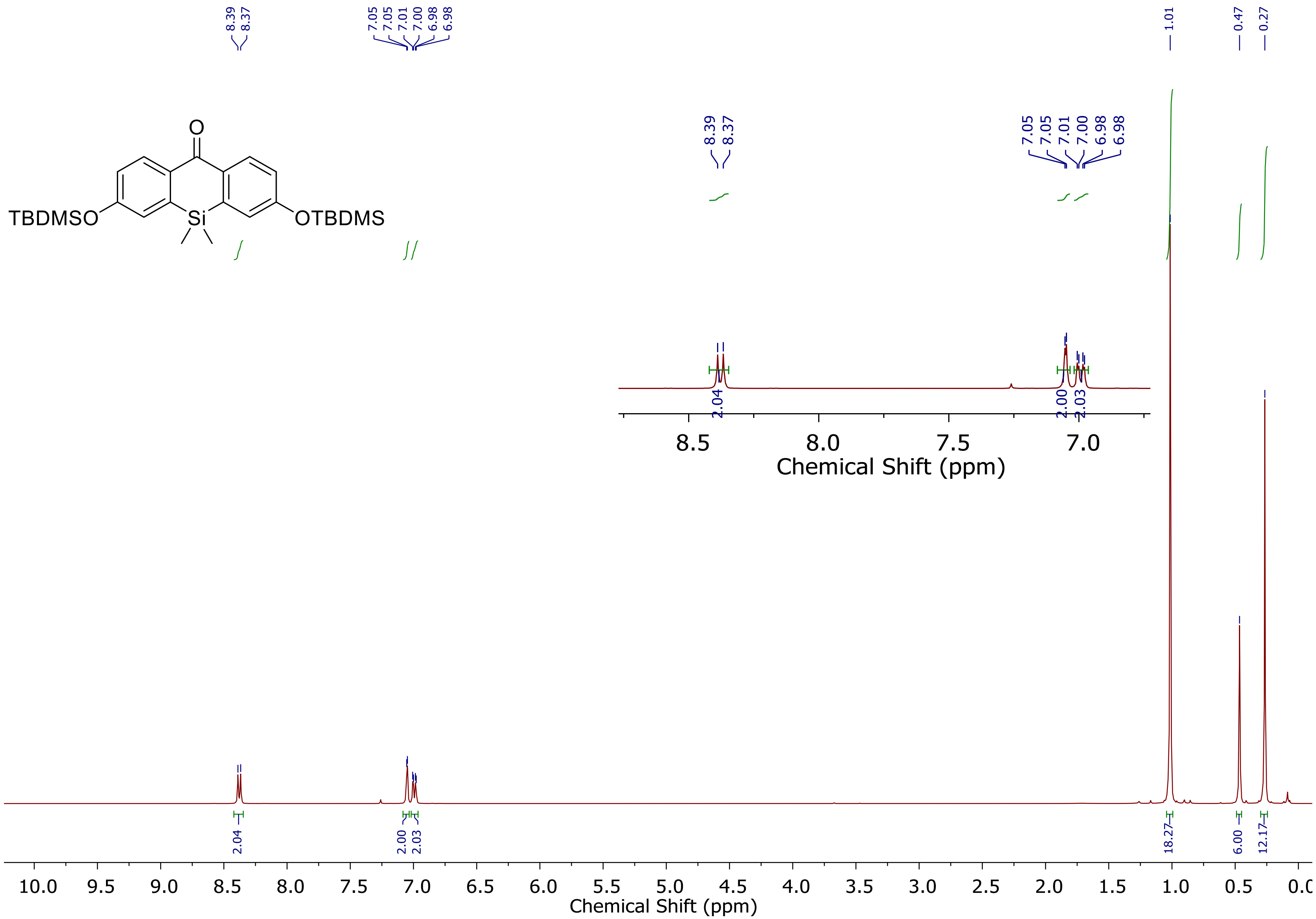

Figure S22. ^1H NMR spectrum of compound **6** in $CDCl_3$.

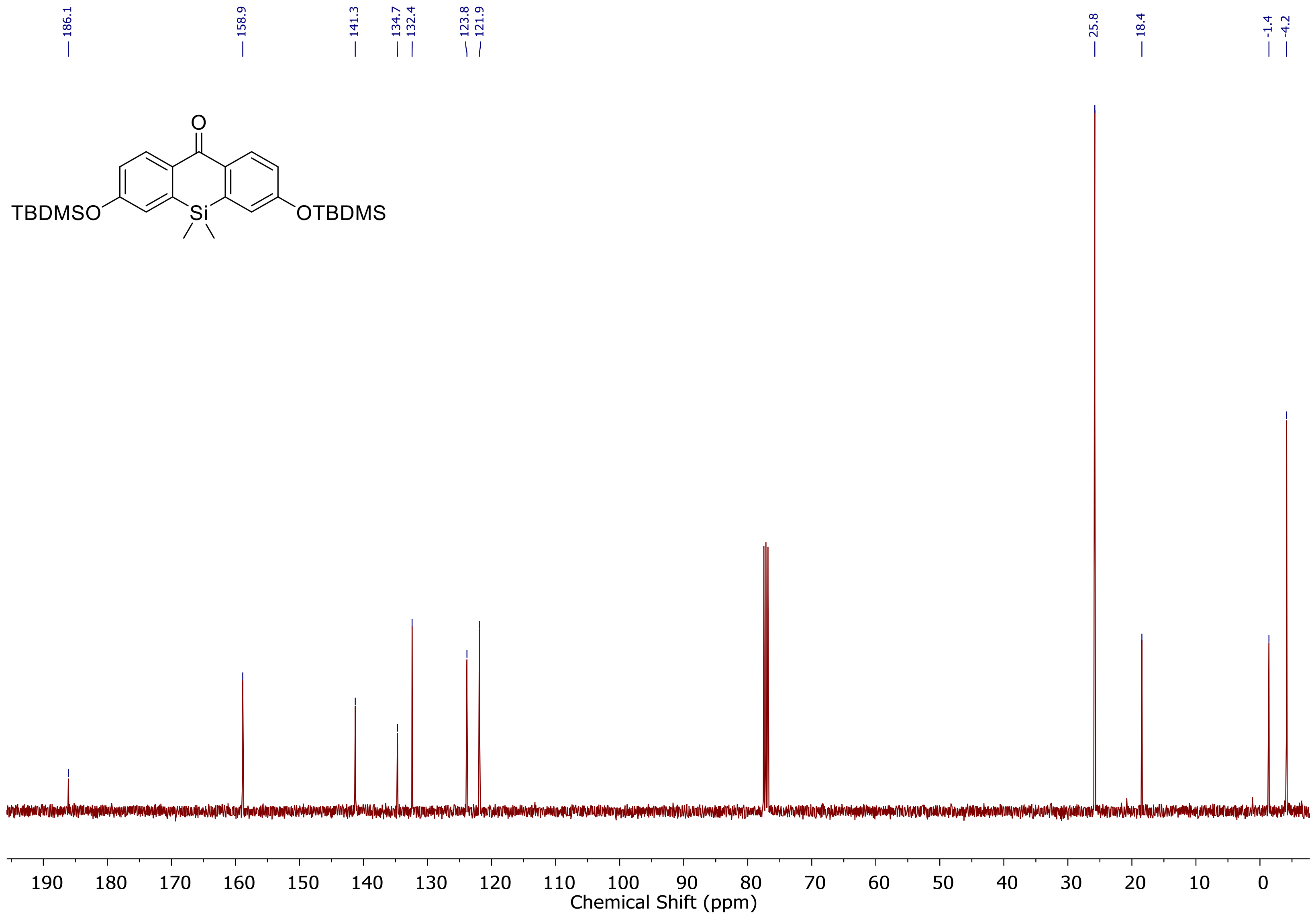

Figure S23. ^{13}C NMR spectrum of compound **6** in $CDCl_3$.

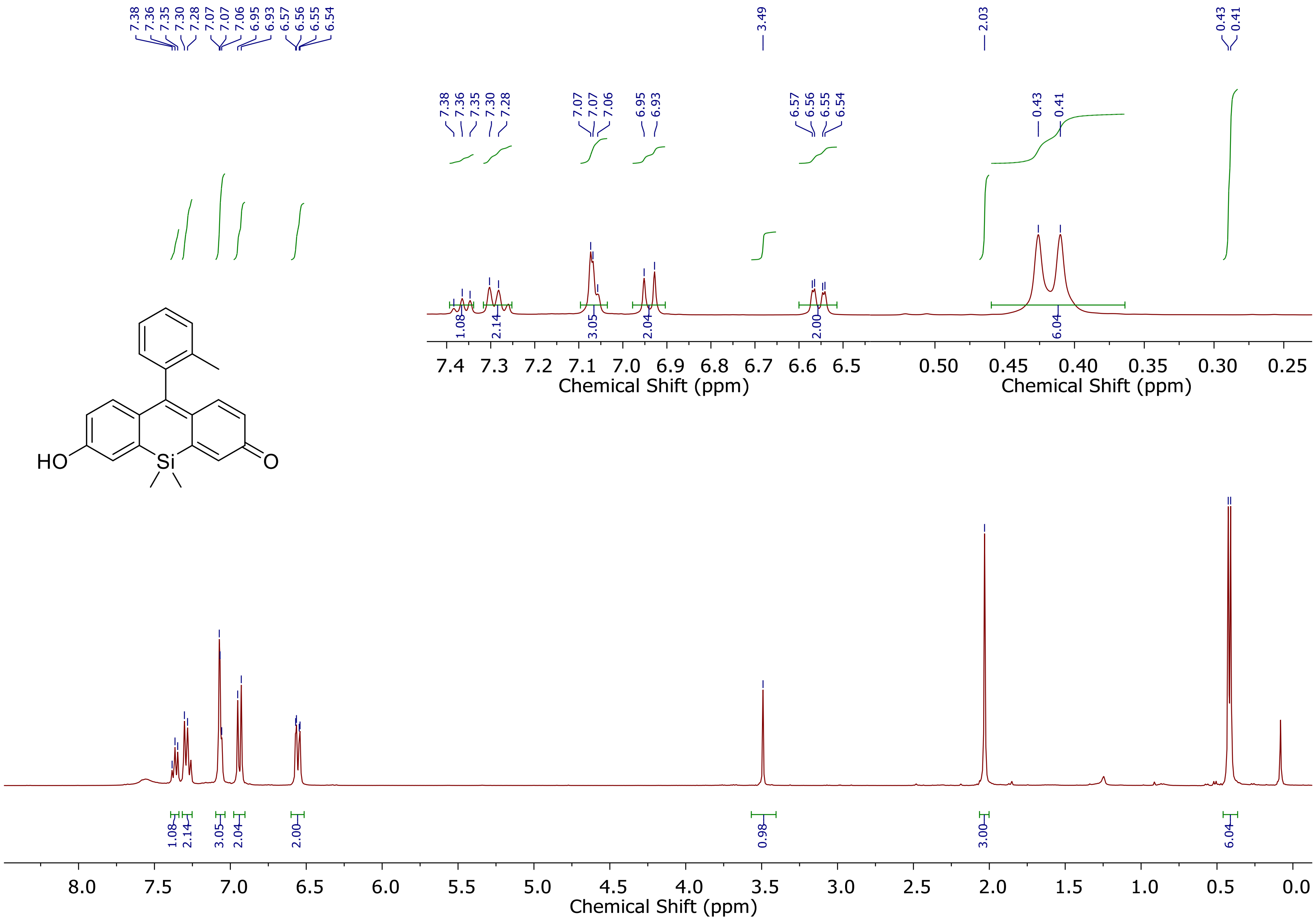

Figure S24. ^{1}H NMR spectrum of compound **7** in $CDCl_3$

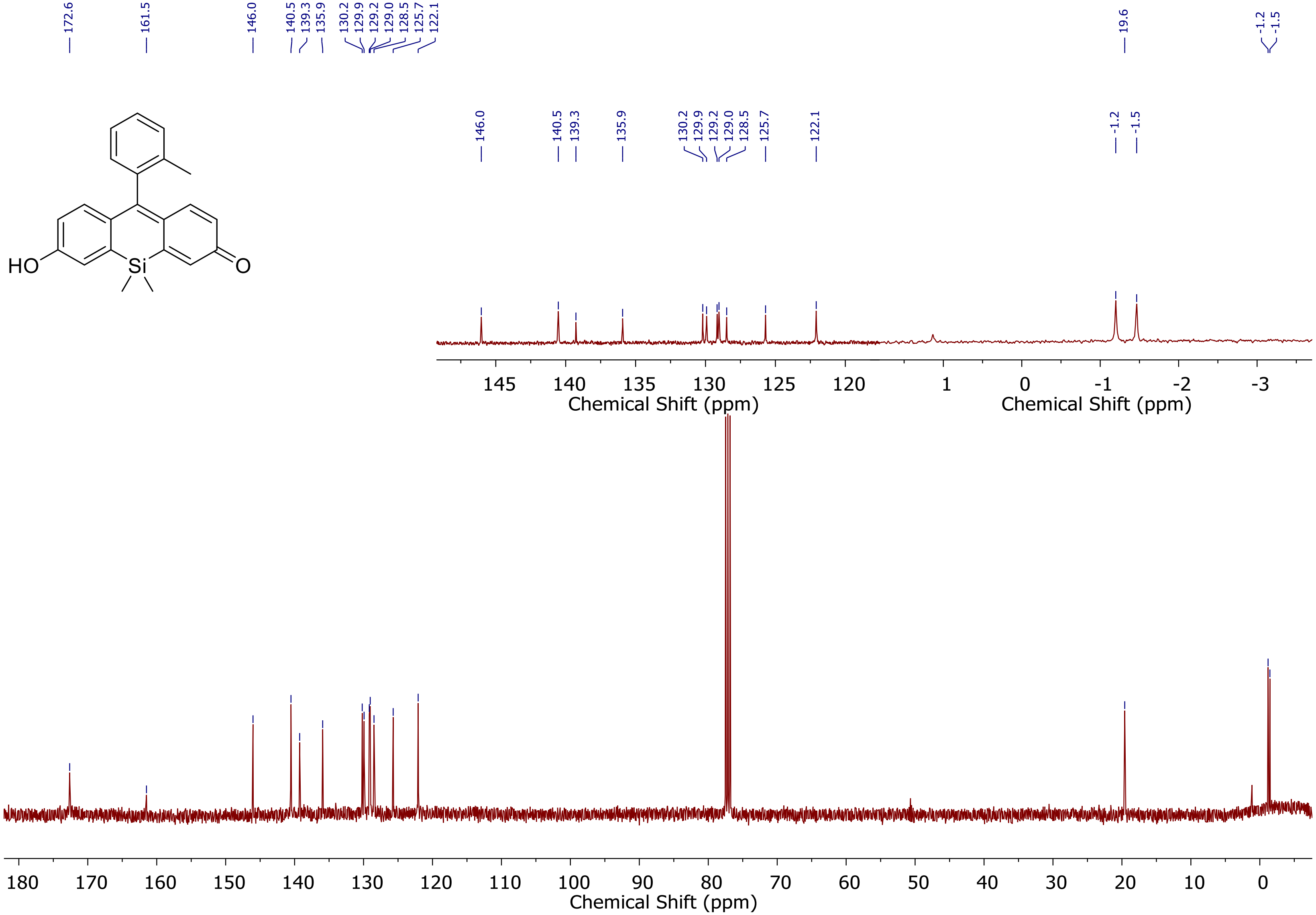

Figure S25. ^{13}C NMR spectrum of compound **7** in $CDCl_3$.

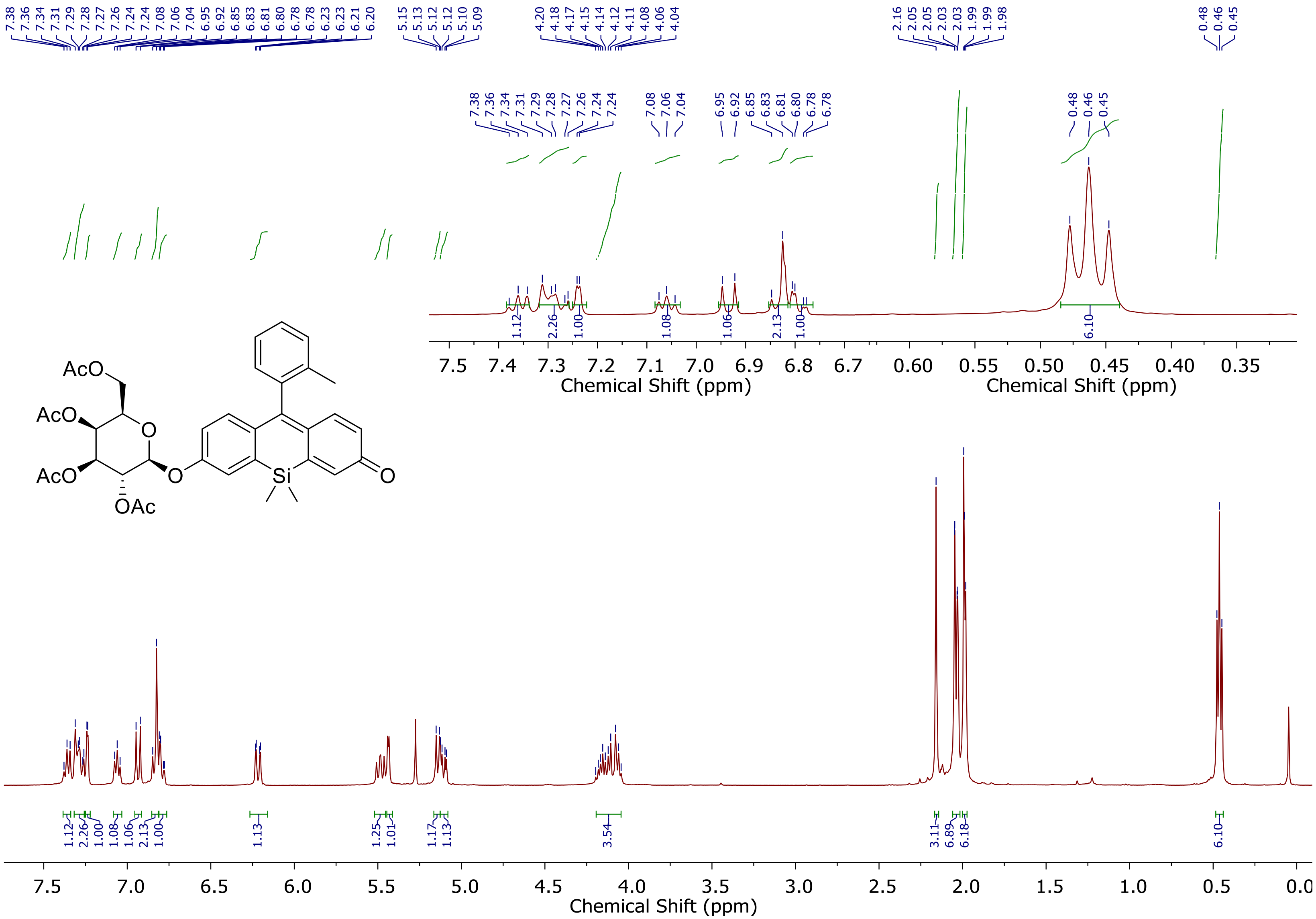

Figure S26. ^{1}H NMR spectrum of compound **8** in $CDCl_3$

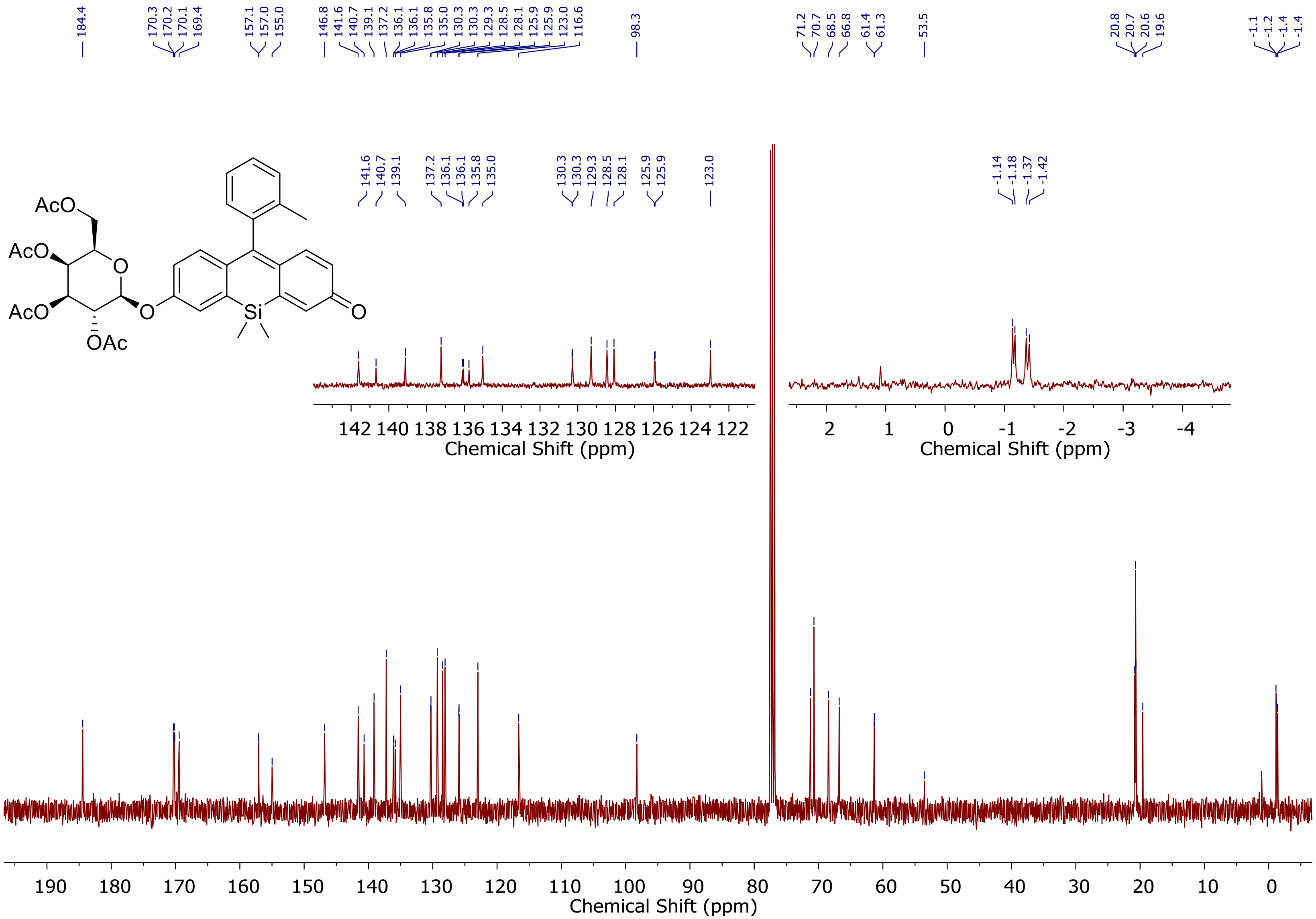

Figure S27. ^{13}C NMR spectrum of compound **8** in $CDCl_3$.

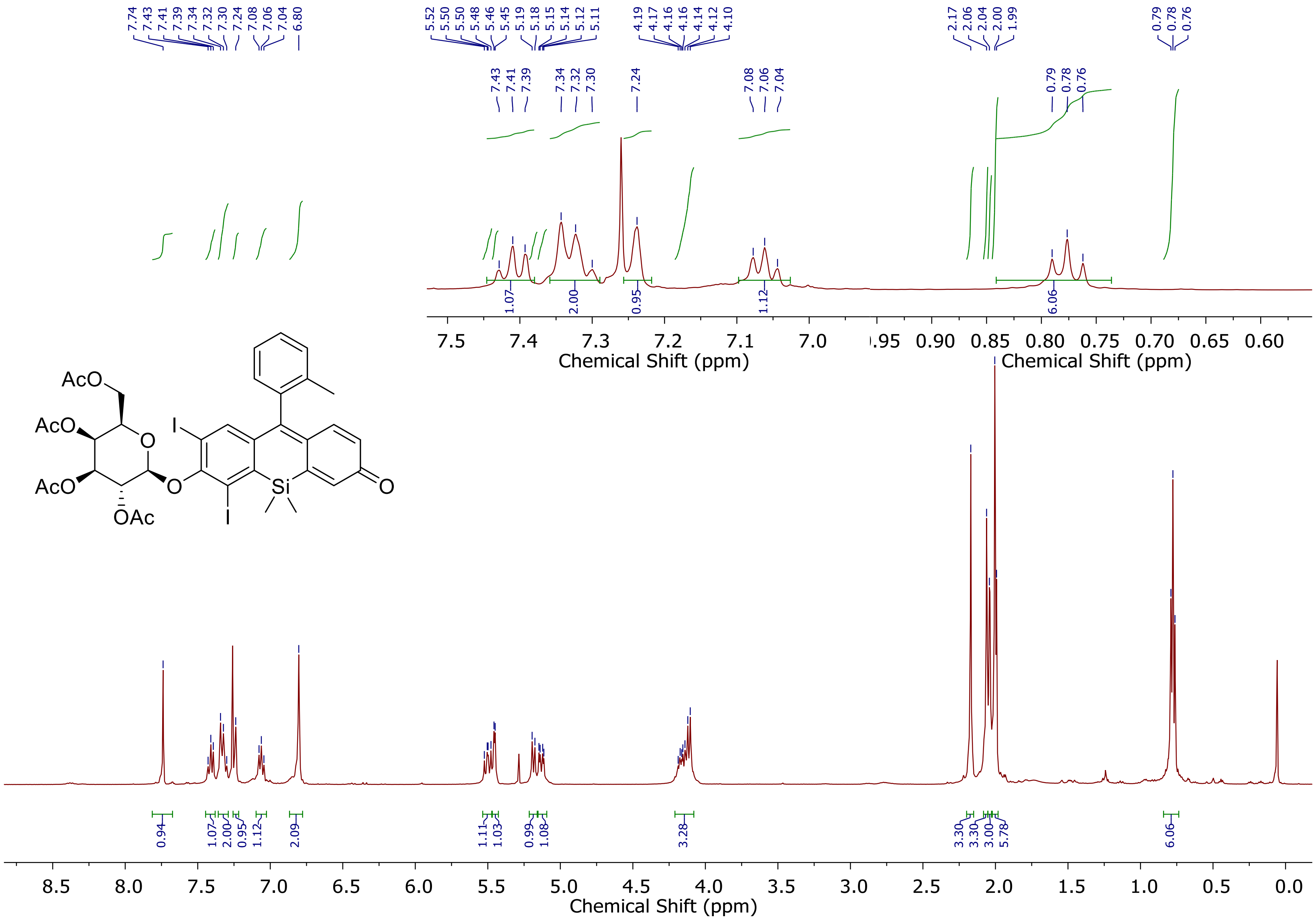

Figure S28. ^{1}H NMR spectrum of compound **9** in $CDCl_3$.

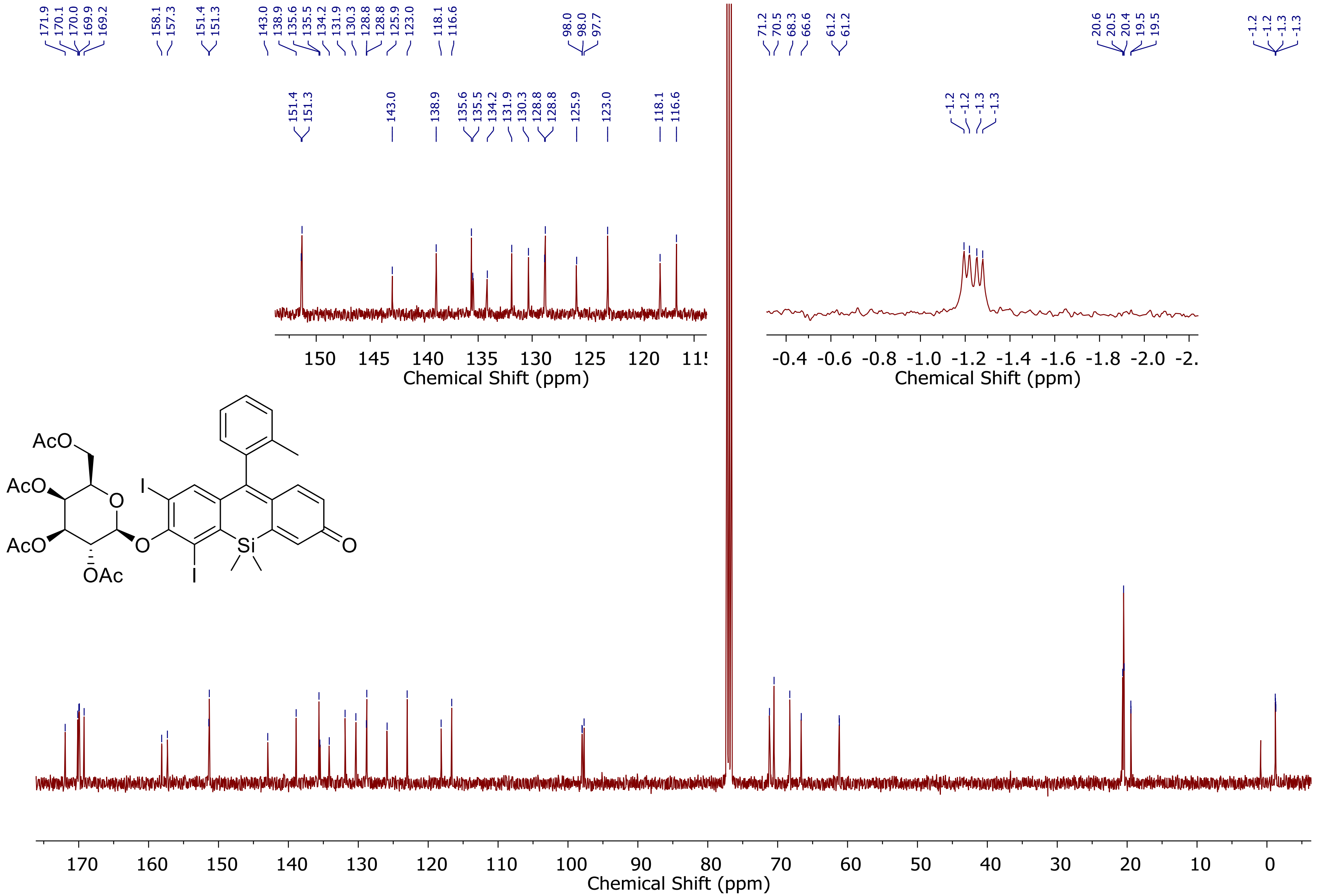

Figure S29. ^{13}C NMR spectrum of compound **9** in $CDCl_3$.

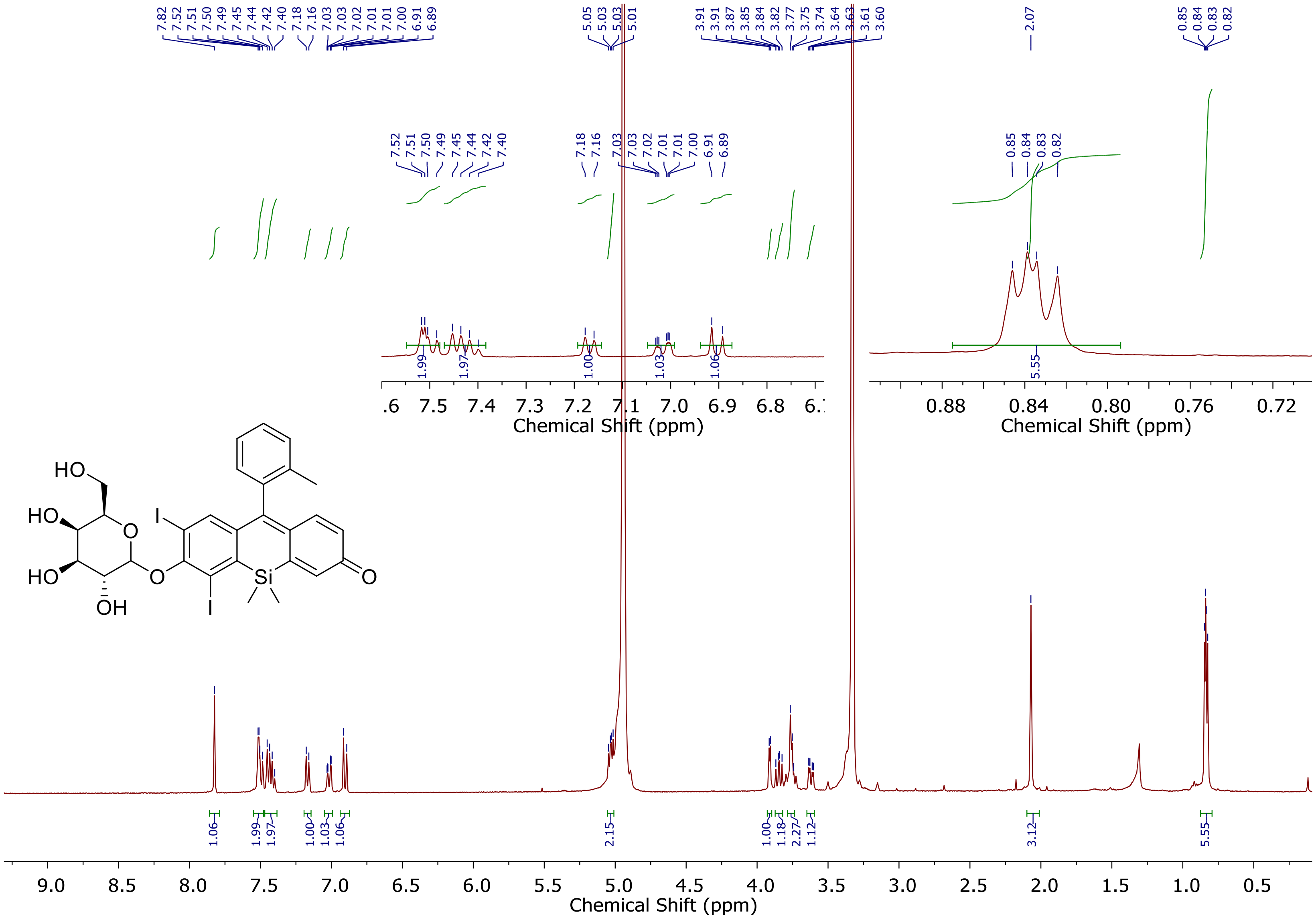

Figure S30. ^{1}H NMR spectrum of **Gal-SiX** in MeOD.

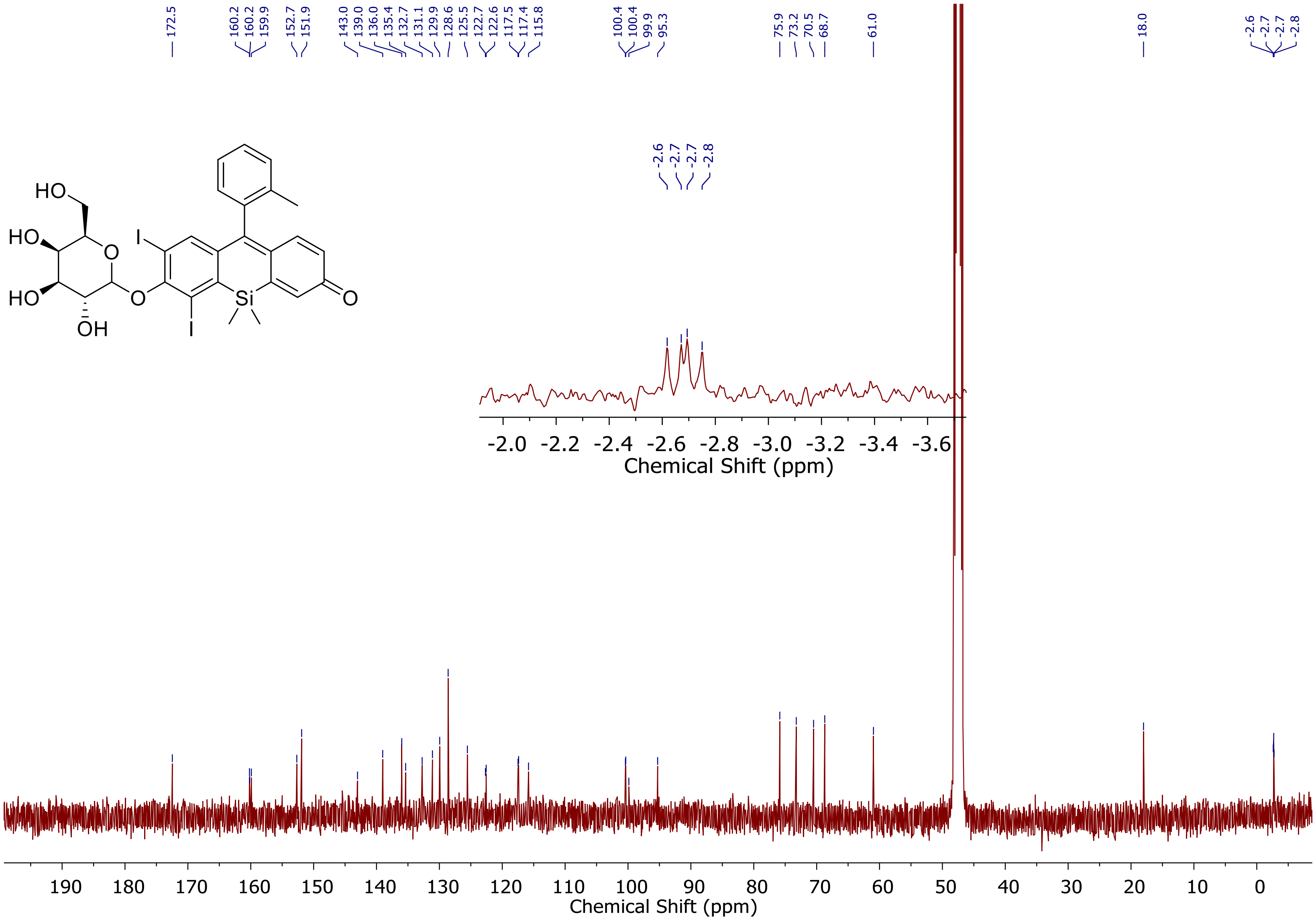

Figure S31. ^{13}C NMR spectrum of **Gal-SiX** in MeOD.

HRMS Spectrum

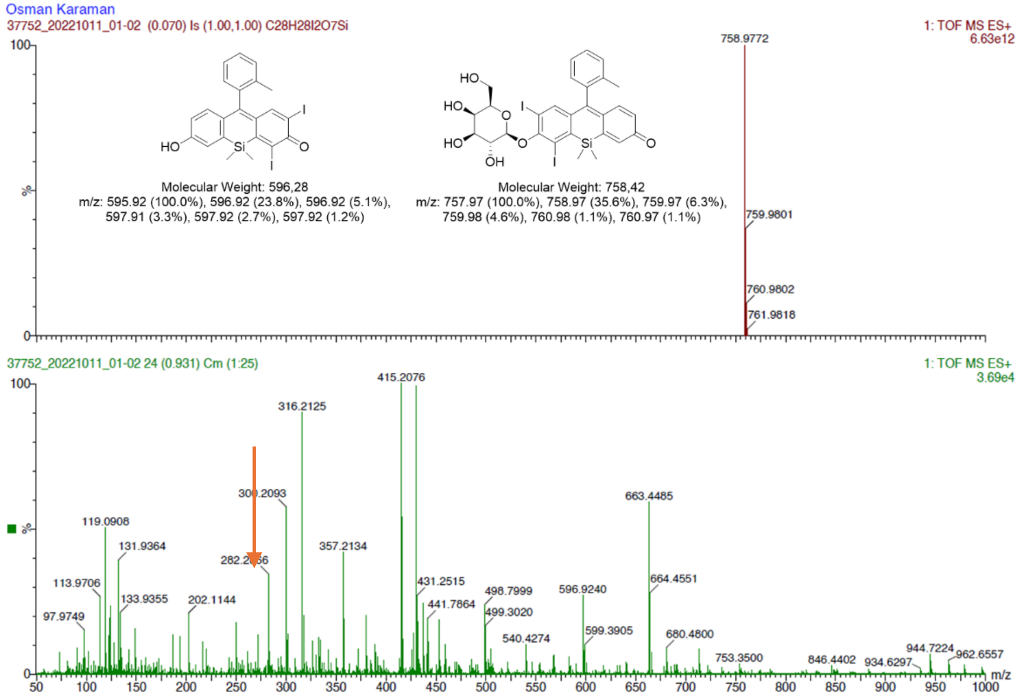

Figure S32. HRMS of **SiF-I**
